# Efficacy and Safety of CAR-T Cell Therapy and Bispecific Antibodies in Relapsed/Refractory Multiple Myeloma with Renal Impairment: A Propensity Score-Matched Analysis

**DOI:** 10.3390/cancers18142311

**Published:** 2026-07-17

**Authors:** Anushareddy Muddasani, Sharmilan Thanendrarajan, Maurizio Zangari, Frits van Rhee, Carolina D. Schinke

**Affiliations:** Myeloma Center, University of Arkansas for Medical Sciences, Little Rock, AR 72205, USAcdschinke@uams.edu (C.D.S.)

**Keywords:** multiple myeloma, CAR-T cell therapy, bispecific antibodies, renal impairment, chronic kidney disease, acute kidney injury, TriNetX, propensity score matching, overall survival, time to next treatment

## Abstract

Background/Objectives: T-cell-redirecting immunotherapies, including chimeric antigen receptor T-cell (CAR-T) therapy and bispecific antibodies (BsAbs), have transformed the treatment of relapsed/refractory multiple myeloma (RRMM). However, pivotal registration trials routinely excluded patients with significant renal impairment (RI), creating a critical evidence gap in a population where kidney disease affects 20–50% of patients at diagnosis. Direct comparisons of outcomes across the estimated glomerular filtration rate (eGFR) spectrum for both modalities are lacking. Methods: We conducted a retrospective cohort study using the TriNetX Global Collaborative Network, a federated electronic health record research platform. Adult patients with RRMM treated with CAR-T therapy (idecabtagene vicleucel or ciltacabtagene autoleucel) or BsAbs (teclistamab, elranatamab, or talquetamab) were stratified by baseline renal function: severe RI (eGFR <30 mL/min/1.73 m^2^), moderate RI (eGFR 30–60 mL/min/1.73 m^2^), and preserved renal function (eGFR >60 mL/min/1.73 m^2^). Propensity score matching (PSM) (1:1), adjusted for key clinical and demographic covariates, was performed for each comparison within each therapy type. Long-term outcomes (all-cause mortality and time to next treatment [TTNT]) were assessed at 1, 2, and 3 years. Overall survival (OS) was additionally evaluated using Kaplan–Meier analysis. Short-term safety outcomes were assessed at 1, 3, and 6 months. Results: A total of 2716 CAR-T and 3376 BsAb recipients were identified. After matching, 281 pairs (severe RI vs. preserved) and 878 pairs (moderate RI vs. preserved) were analyzed in the CAR-T cohort; 645 and 1158 pairs, respectively, were analyzed in the BsAb cohort. Neither severe nor moderate RI was significantly associated with increased mortality or shorter TTNT after either CAR-T or BsAb therapy. However, patients with RI experienced significantly higher rates of anemia, thrombocytopenia, and acute kidney injury (AKI) across both modalities. Cytokine release syndrome (CRS), immune effector cell-associated neurotoxicity syndrome (ICANS), and neutropenia rates were comparable across renal strata. In the BsAb cohort, infections were transiently elevated at 1 month in the severe RI group (RR 1.29; 95% CI 1.06–1.58; *p* = 0.011) but equilibrated by 3 months. Conclusions: In this retrospective analysis, renal impairment was not associated with inferior survival outcomes following CAR-T therapy or BsAb treatment in RRMM, suggesting that RI alone should not preclude the use of these agents. However, RI conferred increased hematologic toxicity and AKI risk, warranting enhanced supportive care and monitoring. These findings support broadening access to T-cell-redirecting immunotherapies for patients with RI with appropriate surveillance, though prospective validation is needed.

## Simple Summary

Kidney disease affects 20–50% of patients with multiple myeloma. Yet, those with significant renal impairment have been routinely excluded from the clinical trials that led to the approval of CAR-T cell therapy and bispecific antibodies. This has created a critical evidence gap regarding the safety and efficacy of these therapies in this common population. Using a large real-world database of over 6000 patients, we compared outcomes across kidney function categories. We found that neither severe nor moderate renal impairment was associated with worse survival or shorter time to next treatment after either therapy. However, patients with impaired kidney function experienced higher rates of anemia, low platelets, and acute kidney injury, highlighting the need for enhanced monitoring and supportive care.

## 1. Introduction

T-cell-redirecting immunotherapies, including chimeric antigen receptor T-cell (CAR-T) therapy and bispecific antibodies (BsAbs), have transformed the treatment landscape for relapsed/refractory multiple myeloma (RRMM), producing deep and durable responses in heavily pretreated patients. Two BCMA (B-cell maturation antigen)-directed CAR-T products, idecabtagene vicleucel (ide-cel) and ciltacabtagene autoleucel (cilta-cel), have demonstrated significant efficacy in registrational and phase 3 trials, and multiple BsAbs targeting BCMA (teclistamab, elranatamab) and G protein-coupled receptor class C group 5 member D (GPRC5D) (talquetamab) have received regulatory approval based on pivotal studies [1,2,3,4,5]. However, the clinical trials that led to the approval of these agents routinely excluded patients with significant renal impairment (RI), with creatinine clearance (CrCl) thresholds typically ranging from 30 to 50 mL/min, creating a critical evidence gap in a population where kidney disease is prevalent [6,7].

Renal impairment is a hallmark complication of multiple myeloma, affecting 20–50% of patients at diagnosis, with frequent recurrence or worsening in the relapsed/refractory setting [6,8,9]. The pathophysiology is multifactorial and involves two distinct, parallel mechanisms of injury. In the distal tubules, overproduction of monoclonal free light chains leads to cast nephropathy through binding with Tamm–Horsfall protein (uromodulin), causing intrarenal obstruction and tubulointerstitial inflammation. Separately, in the proximal tubules, massive reabsorption of free light chains via the megalin/cubilin receptor system activates inflammatory cytokines, oxidative stress, and apoptosis, ultimately resulting in tubulointerstitial fibrosis [9,10]. Additional contributors include hypercalcemia, hyperuricemia, dehydration, high tumor burden, amyloidosis, light chain deposition disease, contrast agent exposure, and nephrotoxic drug exposure [8].

Emerging real-world data have begun to address the evidence gap for CAR-T therapy in renal-impaired patients, though findings have been conflicting. In a retrospective analysis of 28 patients with RI (CrCl <50 mL/min) treated with ide-cel, Sidana et al. reported higher short-term cytopenias, but similar CRS and ICANS rates compared to patients with preserved renal function [11]. Grieb et al. found that CKD status alone did not significantly impact overall survival (OS) or progression-free survival (PFS) in 87 CAR-T recipients; however, a significant interaction between CKD status and CAR-T product was observed, with cilta-cel’s PFS advantage over ide-cel attenuated in patients with moderate/severe CKD [12]. In contrast, other real-world analyses have reported less favorable outcomes. Filippatos et al. found that eGFR <60 mL/min/1.73 m^2^ was associated with significantly higher mortality in 1272 CAR-T recipients [13]. A national analysis by Mahadevia et al. found that CKD stage 3a or higher was associated with markedly increased AKI (58.1% vs. 13.0%; adjusted OR 9.2), higher respiratory failure, and amplified infections, though inpatient mortality, CRS, and ICANS rates were similar [14]. Lymphodepletion strategies also require adaptation in this population, as fludarabine is approximately 40% renally cleared. The European Myeloma Network recommends reducing fludarabine to 24 mg/m^2^ for CrCl 30–70 mL/min; for CrCl <30 mL/min, cyclophosphamide alone or bendamustine (90 mg/m^2^ for 2 consecutive days) represents alternative options [7].

For BsAbs, the available evidence is more reassuring. BsAbs are not eliminated through the kidneys, and dose modifications are generally not required in patients with renal impairment. A systematic review by Ntanasis-Stathopoulos et al. encompassing 11 studies found a comparable overall response rate (ORR), PFS, and OS between patients with and without RI, with only thrombocytopenia significantly increased [15]. Dima et al. reported comparable response rates and survival outcomes in 384 patients treated with teclistamab, with no worsening of renal function in most patients [16]. Case reports have further demonstrated the feasibility of BsAbs in patients with end-stage renal disease on hemodialysis and peritoneal dialysis, with reports of deep responses including stringent complete response and dialysis independence [17,18,19].

While these data collectively suggest that T-cell-redirecting immunotherapies are feasible in patients with RI, the evidence remains fragmented, with conflicting survival data, heterogeneous RI definitions, and a lack of direct cross-modality comparisons. Most studies have examined renal function as a binary variable rather than across a spectrum of GFR categories, limiting the granularity of risk stratification. In this study, we sought to address these gaps by evaluating the efficacy and safety outcomes of both CAR-T therapy and bispecific antibodies across defined eGFR groups (eGFR <30, 30–60, and >60 mL/min/1.73 m^2^) in patients with RRMM using the TriNetX Global Collaborative Network.

## 2. Methods

### 2.1. Study Design and Data Source

This was a retrospective cohort study using the TriNetX Global Collaborative Network, a federated research network providing access to de-identified electronic health record (EHR) data from over 170 participating healthcare organizations across the globe. All cohort queries and analyses were performed between 13 May and 18 May 2026. The TriNetX platform aggregates real-time diagnoses, procedures, medications, laboratory values, and demographic data, enabling large-scale observational research while maintaining compliance with the Health Insurance Portability and Accountability Act (HIPAA). All statistical analyses were conducted using the platform’s built-in analytical tools, which employ Java 11.0.16 (Oracle Corporation, Austin, TX, USA; including Apache Commons Math 3.6.1), R 4.0.2 (R Foundation for Statistical Computing, Vienna, Austria; with Hmisc 4.1-1 and Survival 3.2-3), and Python 3.7 (Python Software Foundation, Wilmington, DE, USA; utilizing lifelines 0.22.4, matplotlib 3.5.1, numpy 1.21.5, pandas 1.3.5, scipy 1.7.3, and statsmodels 0.13.2).

### 2.2. Patient Population

Adult patients (≥18 years) with relapsed or refractory multiple myeloma (RRMM) who received CAR-T cell therapy or bispecific antibody (BsAb) therapy were identified. Multiple myeloma was identified using the International Classification of Diseases, Tenth Revision, Clinical Modification (ICD-10-CM) diagnosis code C90.0. Relapsed or refractory status was defined by the presence of ICD-10-CM codes for multiple myeloma not having achieved remission (C90.00) or multiple myeloma in relapse (C90.02).

### 2.3. Exposure Definitions

CAR-T cell therapy was identified using Current Procedural Terminology (CPT) procedure codes for CAR-T cell administration and medication codes for idecabtagene vicleucel (ide-cel) or ciltacabtagene autoleucel (cilta-cel). BsAb therapy was identified using medication codes (RxNorm), procedure codes, International Classification of Diseases, Tenth Revision, Procedure Coding System (ICD-10-PCS), and Healthcare Common Procedure Coding System (HCPCS) drug administration codes for teclistamab, talquetamab, or elranatamab. The index date was defined as the date of first administration of the qualifying therapy. A complete listing of all codes used in this study is provided in Supplementary Table S1.

### 2.4. Renal Function Stratification

Severe renal impairment was defined as eGFR <30 mL/min/1.73 m^2^ or the presence of diagnosis codes for CKD stage 4 (N18.4), CKD stage 5 (N18.5), end-stage renal disease (N18.6), or dialysis dependence (Z99.2). Moderate RI was defined as eGFR 30–60 mL/min/1.73 m^2^ or a diagnosis code for CKD stage 3 (N18.3), excluding patients meeting severe RI criteria. Preserved renal function was defined as eGFR >60 mL/min/1.73 m^2^, excluding patients with any diagnosis code for CKD stage 3–5, ESRD, or dialysis dependence. Baseline eGFR was defined as the most recent value recorded prior to the index date. The combined approach using both laboratory values and diagnosis codes was employed to maximize capture of renal function status, as neither source alone provides complete ascertainment in EHR data. This combined approach using both laboratory values and diagnosis codes was employed to maximize capture of renal function status.

### 2.5. Propensity Score Matching

Propensity score matching (PSM) was performed separately for each pairwise comparison (severe RI vs. preserved function; moderate RI vs. preserved function) within each therapy type (CAR-T and BsAb), yielding four independent matched analyses. It was performed using the TriNetX Analytics platform’s built-in module, which implements 1:1 greedy nearest-neighbor matching without replacement with a caliper of 0.1 pooled standard deviations of the logit of the propensity score. These parameters are platform-standardized and not user-modifiable. Matching variables included age at index date, sex, race, ethnicity, comorbidities (chronic ischemic heart disease, heart failure, obesity, prior cerebral infarction, COPD, pulmonary fibrosis), prior myeloma-directed therapies (proteasome inhibitors, immunomodulatory agents, anti-CD38 monoclonal antibodies, alkylating/cytotoxic agents), prior autologous stem cell transplantation, and number of prior chemotherapy lines (line 3, 4, 5). Standardized mean differences (SMD) were calculated for all matching variables, with an SMD <0.1 considered indicative of adequate covariate balance.

### 2.6. Outcomes

Long-term efficacy outcomes were assessed at 1, 2, and 3 years from the index date and included overall mortality (death from any cause) and time to next treatment (TTNT), a composite outcome defined as death from any cause or initiation of subsequent myeloma-directed therapy, whichever occurred first. Subsequent myeloma-directed therapy was identified using RxNorm, HCPCS, and ICD-10-PCS codes for proteasome inhibitors, immunomodulatory agents, anti-CD38 monoclonal antibodies, alkylating agents, bispecific antibodies, and CAR-T therapy. For the BsAb cohort, CAR-T therapy was excluded from the TTNT definition, as CAR-T is sometimes administered as planned sequential therapy rather than for disease progression. TTNT was used as a real-world surrogate for progression-free survival, as IMWG-defined response assessments are not available on the TriNetX platform. Short-term safety outcomes were assessed at 1, 3, and 6 months and included CRS, ICANS, AKI (at 1 month only), grade ≥ 3 cytopenias, infections, and hypogammaglobulinemia. Grade ≥ 3 cytopenias were defined using laboratory values: hemoglobin <8.0 g/dL (anemia), platelets ≤ 50 × 10^3^/µL (thrombocytopenia), and absolute neutrophil count < 1.0 × 10^3^/µL (neutropenia).

### 2.7. Statistical Analysis

For binary safety outcomes, risk ratios (RR) with 95% confidence intervals (CI) were calculated. Kaplan–Meier survival estimates were generated for overall survival and TTNT, with between-group differences assessed using the log-rank test. Hazard ratios (HRs) with 95% CIs were estimated from Cox proportional hazards models. A two-sided *p*-value < 0.05 was considered statistically significant. As a sensitivity measure, Benjamini–Hochberg false discovery rate (FDR)–adjusted q-values were calculated for all safety comparisons. To address potential confounding from differential product or agent composition across renal function strata, product-stratified analyses were performed separately for individual CAR-T products and individual bispecific antibody agents. Efficacy outcomes (1-year mortality and TTNT, assessed using Kaplan–Meier estimates with hazard ratios and log-rank tests) and safety outcomes (assessed using risk ratios at 1, 3, and 6 months) were compared between patients with renal impairment (eGFR <30 or eGFR 30–60 mL/min/1.73 m^2^) and those with preserved renal function (eGFR >60 mL/min/1.73 m^2^). All analyses were performed within the TriNetX analytics platform.

## 3. Results

### 3.1. Study Population

A total of 2716 patients receiving CAR-T therapy and 3376 patients receiving BsAb therapy met the inclusion criteria. Within the CAR-T cohort, 1529 patients had preserved renal function, 902 had moderate RI, and 285 had severe RI before matching. Within the BsAb cohort, 1418 patients had preserved renal function; 1266 had moderate RI, and 692 had severe RI before matching. After propensity score matching, the CAR-T therapy cohort comprised 281 matched pairs for the severe RI versus preserved renal function analysis and 878 matched pairs for the moderate RI versus preserved renal function analysis. The BsAb therapy cohort comprised 645 matched pairs for the severe RI analysis and 1158 matched pairs for the moderate RI analysis. Standardized mean differences for all matching variables were below 0.1 after matching, indicating adequate covariate balance across all comparisons. Baseline demographics, comorbidity, and treatment characteristics of the propensity-matched cohorts are summarized in Table 1 (CAR-T) and Table 2 (BsAb).

### 3.2. Efficacy Outcomes

#### 3.2.1. Overall Survival

**Figure 1:**
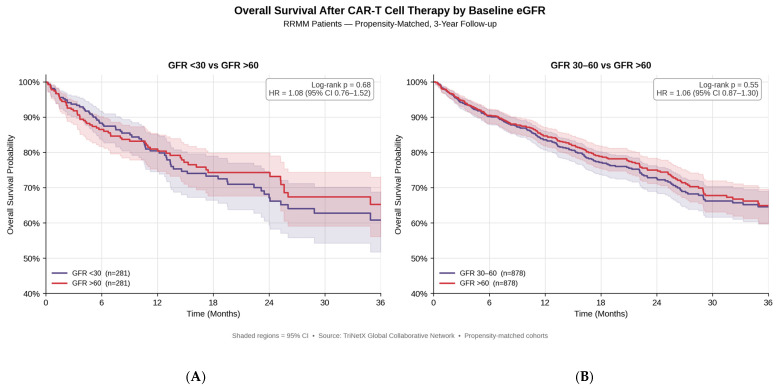
Kaplan–Meier Curves for Overall Survival After CAR-T Cell Therapy. (**A**) eGFR <30 vs. eGFR >60 (*n* = 281 per group). (**B**) eGFR 30–60 vs. eGFR >60 (*n* = 878 per group). Shaded regions represent 95% confidence intervals. HR, hazard ratio; CI, confidence interval.

Neither severe nor moderate renal impairment was associated with significantly worse overall survival after either CAR-T cell therapy or bispecific antibody therapy. In the CAR-T cohort, OS was comparable between patients with severe RI and preserved renal function (HR 1.08, 95% CI 0.76–1.52; log-rank *p* = 0.68), with 1-year survival probabilities of 80.5% versus 80.4% and 3-year survival probabilities of 60.9% versus 65.3%, respectively. The moderate RI comparison was similarly non-significant (HR 1.06, 95% CI 0.87–1.30; log-rank *p* = 0.55), with 1-year survival probabilities of 83.6% versus 84.7%. Median follow-up was 384 versus 429 days in the severe RI comparison and 483 versus 479 days in the moderate RI comparison. Fixed-timepoint mortality outcomes for CAR-T therapy are summarized in Supplementary Table S2. Kaplan–Meier curves for overall survival in the CAR-T cohort, stratified by severe and moderate RI versus preserved renal function, are shown in Figure 1A and Figure 1B, respectively.

**Figure 2:**
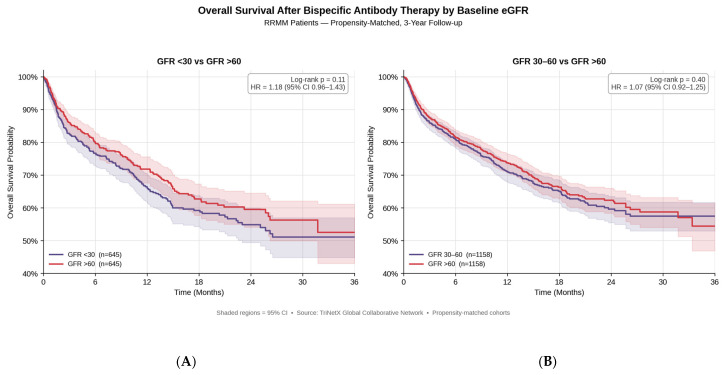
Kaplan–Meier Curves for Overall Survival After Bispecific Antibody Therapy. (**A**) eGFR <30 vs. eGFR >60 (*n* = 645 per group). (**B**) eGFR 30–60 vs. eGFR >60 (*n* = 1158 per group). Shaded regions represent 95% confidence intervals.

In the BsAb cohort, a non-significant trend toward lower OS was observed in the severe RI group (HR 1.18, 95% CI 0.97–1.43; log-rank *p* = 0.11), with 1-year survival probabilities of 66.2% versus 71.8% and 3-year survival probabilities of 51.1% versus 52.5%. The moderate RI comparison showed no difference (HR 1.07, 95% CI 0.92–1.25; log-rank *p* = 0.40), with 1-year survival probabilities of 71.2% versus 73.8%. Median follow-up was 271 versus 278 days in the severe RI comparison and 284 versus 294 days in the moderate RI comparison. Fixed-timepoint mortality outcomes for bispecific antibody therapy are summarized in Supplementary Table S3. Kaplan–Meier curves for overall survival in the bispecific antibody cohort, stratified by severe and moderate RI versus preserved renal function, are shown in Figure 2A and Figure 2B, respectively.

#### 3.2.2. Time to Next Treatment

**Figure 3:**
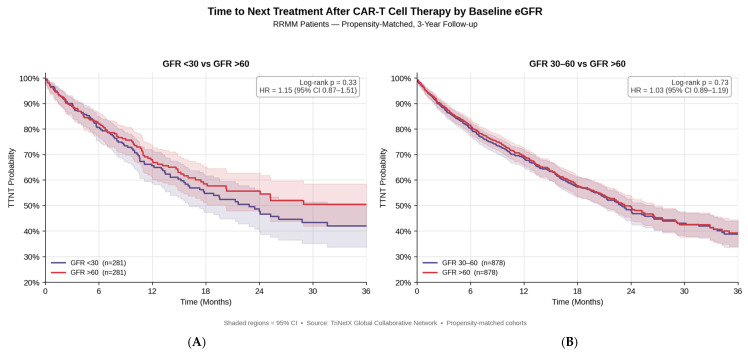
Kaplan–Meier Estimates of Time to Next Treatment After CAR-T Cell Therapy by Renal Function Group. (**A**) eGFR <30 vs. eGFR > 60 (*n* = 281 per group). (**B**) eGFR 30–60 vs. eGFR >60 (*n* = 878 per group). Shaded regions represent 95% confidence intervals.

TTNT was similarly unaffected by renal function status across both modalities. In the CAR-T cohort, no significant difference was observed between severe RI and preserved renal function (HR 1.15, 95% CI 0.87–1.51; log-rank *p* = 0.33; 1-year event-free probability 65.6% vs. 67.4%) or between moderate RI and preserved renal function (HR 1.03, 95% CI 0.89–1.19; log-rank *p* = 0.73; 1-year event-free probability 68.4% vs. 69.3%). Fixed-timepoint TTNT outcomes after CAR-T cell therapy are summarized in Supplementary Table S2. Kaplan–Meier estimates of time to next treatment after CAR-T cell therapy, comparing severe RI (eGFR <30) and moderate RI (eGFR 30–60) versus preserved renal function (eGFR >60), are shown in Figure 3A and Figure 3B, respectively.

**Figure 4:**
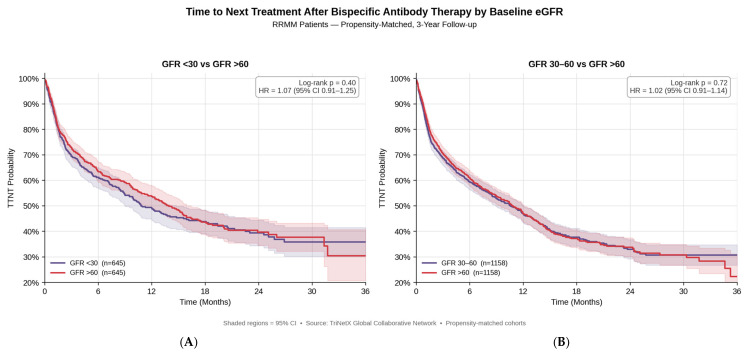
Kaplan–Meier Estimates of Time to Next Treatment After Bispecific Antibody Therapy by Renal Function Group. (**A**) eGFR <30 vs. eGFR > 60 (*n* = 645 per group). (**B**) eGFR 30–60 vs. eGFR >60 (*n* = 1158 per group). Shaded regions represent 95% confidence intervals.

In the BsAb cohort, TTNT was comparable in both the severe RI comparison (HR 1.07, 95% CI 0.91–1.26; log-rank *p* = 0.40; 1-year event-free probability 49.1% vs. 53.7%) and the moderate RI comparison (HR 1.02, 95% CI 0.91–1.14; log-rank *p* = 0.72; 1-year event-free probability 46.8% vs. 46.8%). Fixed-timepoint TTNT outcomes after bispecific antibody therapy are summarized in Supplementary Table S3. Kaplan–Meier estimates of time to next treatment after bispecific antibody therapy, comparing severe RI (eGFR <30) and moderate RI (eGFR 30–60) versus preserved renal function (eGFR >60), are shown in Figure 4A and Figure 4B, respectively.

A summary of efficacy outcomes across all comparisons is presented in Table 3.

### 3.3. Safety Outcomes

Adverse events were assessed at 1, 3, and 6 months across all four matched comparisons. Several consistent patterns emerged across modalities and renal function strata, summarized below by outcome domain.

#### 3.3.1. Hematologic Toxicity

Grade ≥ 3 anemia was the most consistently elevated adverse event, reaching statistical significance in all four comparisons at all assessed timepoints. Effect sizes demonstrated a severity-dependent relationship with baseline renal function: risk ratios ranged from 1.11 to 1.15 in the moderate RI comparisons to 1.26–1.46 in the severe RI comparisons, with the largest effect observed in the BsAb severe RI group (RR 1.46 at 1 month; *p* < 0.001). Grade ≥ 3 thrombocytopenia was significantly elevated in both severe RI comparisons (CAR-T: RR 1.17–1.21; BsAb: RR 1.21–1.26; all *p* < 0.05) but was not significantly different in either moderate RI comparison. Grade ≥ 3 neutropenia was comparable across all renal strata in both modalities. In the CAR-T cohort, neutropenia rates were uniformly high (85–92%) regardless of renal function, reflecting the myelosuppressive effect of fludarabine/cyclophosphamide lymphodepletion conditioning. Hypogammaglobulinemia was numerically elevated in the CAR-T severe RI group but reached statistical significance only at 6 months (RR 1.19; *p* = 0.04); rates were comparable in all other comparisons.

#### 3.3.2. Acute Kidney Injury

AKI at 1 month was significantly elevated in three of four comparisons, with effect sizes that increased with the severity of baseline renal impairment. The largest signal was observed in the BsAb severe RI group (34.1% vs. 13.6%; RR 2.50, 95% CI 2.00–3.12; *p* < 0.001). In the moderate RI comparisons, AKI was significantly elevated in both the CAR-T cohort (19.1% vs. 13.9%; RR 1.38; *p* = 0.003) and the BsAb cohort (20.7% vs. 12.2%; RR 1.70; *p* < 0.001). In the CAR-T severe RI comparison, AKI showed a borderline elevation that did not reach statistical significance (18.9% vs. 13.2%; RR 1.43; *p* = 0.07).

#### 3.3.3. Infections

All-grade infection rates were comparable between renal function groups in both moderate RI comparisons across all timepoints and both modalities. In the severe RI comparisons, infections were numerically higher in both the CAR-T and BsAb cohorts, but statistical significance was reached only in the BsAb severe RI group at 1 month (26.7% vs. 20.6%; RR 1.29, 95% CI 1.06–1.58; *p* = 0.011). This difference attenuated to non-significance by 3 months (RR 1.09; *p* = 0.27) and 6 months (RR 1.08; *p* = 0.26).

#### 3.3.4. CRS and ICANS

Rates of CRS and ICANS were comparable across all renal function strata in all four comparisons at 1 month, with no significant differences observed for either modality.

#### 3.3.5. Interpretation of Safety Findings

Findings that were consistent across multiple comparisons and time points with *p* < 0.001 (grade ≥ 3 anemia, AKI) were also the most statistically robust to correctio for multiple testing (Section 3.5), whereas findings reaching significance at isolated timepoints (e.g., hypogammaglobulinemia at 6 months in the CAR-T severe RI group) did not survive correction and should be interpreted with caution. Forest plots for safety outcomes across all four renal-function comparisons are provided in Supplementary Figures S1–S4.

A comprehensive summary of safety outcomes across all four propensity-matched comparisons, including risk ratios with 95% confidence intervals at 1, 3, and 6 months, is presented in Table 4.

### 3.4. Product-Stratified Subgroup Analyses

To evaluate whether the efficacy and safety patterns observed in the pooled analyses were consistent across individual agents, product-stratified analyses were performed for both CAR-T products and all three bispecific antibody agents. For efficacy, no individual CAR-T product or bispecific antibody showed a statistically significant difference in 1-year mortality or TTNT across renal function strata, confirming that a single agent did not drive the non-significant survival findings in the pooled analyses. Product-stratified 1-year mortality and TTNT outcomes for individual CAR-T products and bispecific antibody agents are presented in Supplementary Tables S4 and S5, respectively.

For safety, product-level analyses supported the pooled toxicity signals. In the CAR-T cohort, significant increases in grade ≥ 3 anemia and thrombocytopenia were most evident with cilta-cel in the severe RI group. Ide-cel showed directionally concordant trends that did not reach statistical significance, likely reflecting smaller subgroup sample sizes and platform-mandated cell suppression rather than a true absence of effect. In the moderate RI comparison, neither CAR-T product individually reached statistical significance for these outcomes. CRS and ICANS rates remained comparable across renal strata for both products. Product-stratified safety outcomes for individual CAR-T products are detailed in Supplementary Table S6.

In the BsAb cohort, teclistamab and talquetamab showed consistent increases in AKI and grade ≥ 3 anemia and thrombocytopenia across renal strata, whereas elranatamab estimates were directionally similar but underpowered due to a smaller sample size. CRS and ICANS rates were comparable across renal strata for all three agents. Product-stratified safety outcomes for individual bispecific antibody agents are detailed in Supplementary Table S7.

These findings indicate that the pooled analyses provide the most stable estimates, while product-stratified results demonstrate broadly consistent safety patterns across agents, mitigating the concern that differential product composition across eGFR strata confounded the primary results.

### 3.5. Multiple Testing Correction for Safety Outcomes

Given the number of safety comparisons performed across outcomes, timepoints, and renal-function strata (*n*  =  72 pairwise tests spanning CRS, ICANS, AKI, grade ≥ 3 anemia, grade ≥ 3 thrombocytopenia, grade ≥ 3 neutropenia, infections, and hypogammaglobulinemia in Table 4), the Benjamini–Hochberg procedure was applied to control the false discovery rate (FDR) at 5% within this safety-outcome family. Raw *p*-values and FDR-adjusted q-values for all 72 safety comparisons are provided in Supplementary Table S8.

Of the 23 comparisons significant at raw *p*  <  0.05, 17 remained significant after FDR correction (q  <  0.05). AKI was the most robust safety signal, remaining significant in the BsAb severe RI comparison (q  =  0.008), the BsAb moderate RI comparison (q  =  0.008), and the CAR-T moderate RI comparison (q  =  0.024). Grade ≥ 3 anemia was similarly robust, remaining significant across all three timepoints in the CAR-T severe RI, BsAb severe RI, and CAR-T moderate RI comparisons (q  =  0.008–0.034). Grade ≥ 3 thrombocytopenia remained significant after correction in the CAR-T severe RI comparison at 3 and 6 months and the BsAb severe RI comparison at 1 and 6 months, and the 1-month infection signal in the BsAb severe RI group narrowly survived correction (q  =  0.047).

Comparisons that were nominally significant but did not survive FDR correction. These include grade ≥ 3 thrombocytopenia in the CAR-T severe RI group at 1 month and the BsAb severe RI group at 3 months, grade ≥ 3 anemia in the BsAb moderate RI group at all three timepoints, and hypogammaglobulinemia in the CAR-T severe RI group at 6 months. These are considered exploratory findings that warrant confirmation in future cohorts rather than robust safety signals. Together, these results indicate that the two principal safety conclusions of this study, the AKI signal and the grade ≥ 3 anemia signal, are the most statistically robust findings and are not attributable to chance alone from the number of comparisons performed.

## 4. Discussion

Our principal finding is that neither severe (eGFR <30) nor moderate (eGFR 30–60) renal impairment was associated with significantly increased mortality or shorter time to next treatment after either CAR-T or BsAb therapy compared to patients with preserved renal function (eGFR >60), after propensity score matching for key demographic, comorbidity, and treatment-related variables. However, renal impairment was consistently associated with increased hematologic toxicity, particularly grade ≥ 3 anemia and thrombocytopenia, and a severity-dependent increase in AKI risk across both therapeutic modalities. Product-stratified subgroup analyses confirmed that these findings were not driven by differential agent composition across renal strata. Neither ide-cel nor cilta-cel individually showed significant differences in 1-year mortality or TTNT by renal function, and the same was true for individual BsAbs. This consistency across agents with different targets, approval timelines, and pharmacologic properties strengthens the generalizability of the pooled efficacy findings.

These findings have direct implications for treatment access, risk stratification, and supportive care planning in a population that has been underrepresented in clinical trials.

The absence of a mortality difference in our CAR-T cohort is concordant with prior smaller studies. Sidana et al. and Grieb et al. both reported preserved survival outcomes among renally impaired CAR-T recipients, though in substantially smaller cohorts (28 and 87 patients, respectively) [11,12]. Our study extends these findings by demonstrating that, after comprehensive propensity matching across a larger population with granular GFR stratification, the mortality signal remains non-significant even at 3 years.

For BsAbs, our results similarly confirm and extend the findings of Ntanasis-Stathopoulos et al. and Dima et al., who reported comparable efficacy and survival in renally impaired patients in smaller cohorts [15,16]. Our study demonstrates that these favorable outcomes persist across a granular GFR spectrum in a substantially larger propensity-matched population, and that the safety profile, including the absence of excess CRS, ICANS, or neutropenia, is consistent across renal strata. The non-significant trend toward higher 1-year mortality in the BsAb severe RI group that attenuated over longer follow-up likely reflects early non-treatment-related mortality from comorbidities in this older, more comorbid population rather than a treatment-specific effect.

The hematologic toxicity signal was consistent across both modalities and demonstrated a dose–response relationship with renal impairment severity. Grade ≥ 3 anemia was the most prominent finding, with risk ratios ranging from 1.11 to 1.46 depending on the degree of RI and therapeutic modality. This likely reflects the convergence of baseline renal anemia due to impaired erythropoietin production and treatment-related myelosuppression. The persistence of this signal through 6 months suggests that renal anemia is a sustained contributor rather than a transient peri-treatment phenomenon. Grade ≥ 3 thrombocytopenia followed a similar but more selective pattern, reaching significance only in the severe RI comparisons for both modalities. A systematic review of 1117 BsAb-treated patients similarly identified thrombocytopenia as the only hematologic toxicity significantly increased in renally impaired patients [15], and higher short-term cytopenias were reported in a cohort of 28 CAR-T recipients with CrCl < 50 mL/min [11]. These findings collectively underscore the importance of proactive transfusion support and close hematologic monitoring in renally impaired patients receiving either modality.

AKI demonstrated a clear severity-dependent relationship with baseline renal impairment severity across both modalities. The highest AKI rates were observed in the BsAb severe RI group (34.1% vs. 13.6%; RR 2.50; *p* < 0.0001). Notably, our findings contrast with those of Chen et al., who reported that baseline tumor burden rather than baseline renal function, was the independent risk factor for AKI in a single-center analysis of 111 CAR-T recipients with RRMM. CRS and ICANS incidence were similar between AKI and non-AKI groups in that study. Our propensity-matched analysis, which controlled for comorbidities and treatment history, demonstrates that lower baseline eGFR itself confers increased AKI risk independent of these factors, suggesting that pre-existing renal vulnerability is an important and potentially underrecognized contributor [20]. A retrospective comparison of teclistamab and CAR-T recipients similarly found that elevated baseline free light chain levels were significantly associated with AKI incidence in the teclistamab group, implicating paraprotein-mediated renal injury and disease progression as additional contributors [21]. Differences in treatment exposure patterns may also contribute; unlike the one-time infusion of CAR-T cells, BsAbs require continuous dosing with repeated T-cell activation, which could result in cumulative hemodynamic perturbations in patients with limited renal reserve, though this hypothesis remains unproven.

Multiple mechanisms of nephrotoxicity have been described in T-cell-engaging BsAb recipients, including CRS-mediated capillary leak and hypotension leading to prerenal AKI, cytokine-mediated inflammatory glomerular injury, tumor lysis syndrome with crystal-induced tubular obstruction, and sepsis-related renal damage [22]. The interplay between these mechanisms may be amplified in patients with pre-existing renal vulnerability. Specifically, patients with severe RI have diminished nephron reserve and reduced tolerance to hemodynamic perturbations during CRS episodes, while CKD-associated immune dysfunction may predispose to infections that independently cause AKI. Further studies are needed to delineate the relative contributions of disease progression, direct drug toxicity, and treatment exposure patterns to AKI in this population.

Infection rates were comparable between RI and preserved renal function groups in both moderate RI comparisons across all timepoints and both modalities. In contrast, patients with severe RI demonstrated numerically higher infection rates than their matched references in both the CAR-T and BsAb cohorts, though statistical significance was reached only in the BsAb severe RI group at 1 month (RR 1.29; *p* = 0.011), equilibrating by 3 months. CKD is associated with a state of immune dysfunction characterized by chronic low-grade activation of innate immune cells alongside paradoxically impaired antimicrobial capacity [23]. Uremic toxins accumulate in advanced CKD and directly modulate immune cell function, contributing to both inflammatory activation and immunosuppression [24]. When T-cell-redirecting immunotherapies are superimposed on this baseline immunodeficiency, the result may be a compounded vulnerability. A systematic review and meta-analysis of infections following BsAbs in myeloma reported a pooled all-grade infection prevalence of 56% and grade ≥ 3 infection prevalence of 24% [25]. Similarly, a meta-analysis of nonrelapse mortality after CAR-T therapy found that infections accounted for over half of all nonrelapse deaths [26]. These findings support enhanced infection surveillance and prophylaxis in patients with eGFR <30 receiving T-cell-redirecting therapies [27,28,29].

Neutropenia rates in the CAR-T cohort were uniformly high across all renal strata (85–92%) and showed no difference by baseline renal function, in contrast to the BsAb cohort where neutropenia rates were substantially lower (20–42%) and similarly unaffected by renal status. The near-universal neutropenia in CAR-T recipients is consistent with the registrational trial data for both ide-ce; and cilta-cel reflecting the predictable myelosuppressive effect of fludarabine/cyclophosphamide lymphodepletion conditioning rather than any renal function-dependent mechanism. The lower neutropenia rates in BsAb recipients are expected given the absence of lymphodepletion conditioning in BsAb protocols.

The observation that CRS and ICANS rates were comparable across renal strata for both modalities is clinically reassuring and consistent with the published literature. For CAR-T therapy, preserved CRS and ICANS profiles in renally impaired patients have been reported in a single-center ide-cel cohort (*n* = 28) [11], a multicenter analysis of 87 CAR-T recipients [12], and a national analysis using the National Inpatient Sample encompassing over 2000 weighted CAR-T hospitalizations [14]. Our study extends these findings by demonstrating that CRS and ICANS rates remain comparable even in patients with severe RI (eGFR <30), a population not separately analyzed in prior studies. For BsAb therapy, comparable CRS and ICANS rates between renally impaired and non-impaired teclistamab recipients were reported in a multicenter analysis of 384 patients [16] and a systematic review encompassing 1117 patients across multiple studies [15]. These consistent findings suggest that baseline renal function does not fundamentally alter the T-cell activation kinetics or cytokine release dynamics that drive these toxicities with either modality.

The implications of these findings for clinical practice are several. First, renal impairment should not be considered a contraindication to either CAR-T therapy or BsAbs in RRMM. The preserved efficacy across the GFR spectrum supports broadening access to these therapies, consistent with the International Myeloma Working Group recommendations [6]. Second, the choice between CAR-T and BsAb therapy in renally impaired patients should be guided by the same factors that inform this decision in the general RRMM population rather than by renal function alone [30]. Third, enhanced supportive care is warranted, including proactive transfusion support, erythropoiesis-stimulating agents, AKI prevention strategies, and heightened infection surveillance. Fourth, for CAR-T therapy in patients with severe RI, lymphodepletion regimen adaptation is essential per European Myeloma Network recommendations [7]. Case reports of successful BsAb use in patients on hemodialysis and peritoneal dialysis further support the feasibility of T-cell-redirecting therapies even in end-stage renal disease [17,18,19].

## 5. Limitations

This study has several limitations inherent to its retrospective, EHR-based design. First, the TriNetX platform relies on ICD-10-CM diagnosis codes and laboratory values for outcome ascertainment, which may be subject to coding variability and misclassification. Standard IMWG-defined response assessments (overall response rate, complete response, very good partial response) are not available in TriNetX, necessitating the use of TTNT as a real-world surrogate for PFS. TTNT remains an imperfect surrogate for PFS, as treatment switches driven by toxicity or planned sequencing rather than disease progression may trigger events. To mitigate this, CAR-T therapy was excluded from the BsAb cohort’s TTNT definition, as CAR-T is sometimes administered as planned sequential therapy rather than for disease progression. Any residual misclassification would apply equally across eGFR strata. AKI was assessed at 1 month only. While this captures the highest-risk period for treatment-related AKI, it may underestimate cumulative renal events in BsAb recipients receiving continuous dosing. However, extending the AKI window would risk conflating treatment-related AKI with renal deterioration from disease progression, intercurrent illness, or nephrotoxic exposures, as ICD-10-CM codes do not distinguish AKI etiology. Additionally, the analysis did not capture infection prophylaxis practices across eGFR strata. TriNetX records medication administration but cannot reliably distinguish prophylactic from therapeutic use, and medication timing relative to the index event is not granular enough to make this determination. Second, the renal function stratification combined eGFR values and ICD-10-CM diagnosis codes, which may introduce heterogeneity within groups. The eGFR value used reflects the most recent measurement prior to the index date, which in some patients may capture acute fluctuations rather than stable baseline renal function. The severe RI group encompasses a spectrum from CKD stage 4 to dialysis-dependent ESRD, and the platform does not permit reporting the proportion classified by laboratory values alone versus diagnosis codes alone. Any misclassification from coding inconsistencies would occur independently of patient outcomes, making the groups appear more similar and biasing results toward finding no difference rather than creating a false signal. Third, although propensity score matching achieved adequate balance for measured covariates, unmeasured confounders including cytogenetic risk (del(17p), t(4;14), gain(1q)), International Staging System stage, serum free light chain levels, tumor burden, and BCMA expression could not be accounted for. These unmeasured variables may influence both treatment outcomes and renal function trajectories, and their absence limits causal inference. The number of prior therapy lines was proxied using platform-derived VA Class codes for chemotherapy lines 3, 4, and 5. While these codes are imprecise proxies for true myeloma-specific treatment lines, individual prior drug class exposures and prior autologous stem cell transplantation were also included as matching variables and achieved adequate balance (all SMD <0.1). Nonetheless, residual confounding related to the true number and composition of prior treatment lines cannot be excluded. The elevated AKI rates observed in renally impaired patients may therefore partly reflect residual confounding by disease burden rather than treatment-related nephrotoxicity alone. ECOG performance status, which may be worse in patients with severe renal impairment, was sparsely documented in TriNetX and could not be reliably adjusted for across the full cohort; residual confounding by functional status therefore cannot be excluded. Additionally, temporal trends in treatment adoption and evolving supportive care practices over the study period (2021–2026) could introduce confounding not fully addressed by propensity score matching; however, the narrow treatment era and matching on prior therapy lines partially mitigate this concern. TriNetX does not capture drug dosing or lymphodepletion regimen details; therefore, whether fludarabine dose reductions or alternative conditioning regimens were used in renally impaired CAR-T recipients could not be assessed, though the comparable grade ≥ 3 neutropenia rates across all eGFR strata provide indirect evidence of similar lymphodepletion intensity. Product-stratified safety and efficacy analyses were performed. While individual-agent subgroups were smaller and some comparisons were underpowered, the direction of effect was broadly consistent across agents for both efficacy and safety outcomes. Fourth, the relatively short median follow-up for the BsAb cohort (approximately 9 months) compared to the CAR-T cohort (approximately 13–16 months) limits the reliability of longer-term estimates, particularly at 2 and 3 years where progressively fewer patients remained at risk. Furthermore, the TriNetX platform does not display the number of patients at risk at each timepoint on Kaplan–Meier curves, limiting the reader’s ability to assess the precision of later estimates. The 2- and 3-year BsAb estimates should therefore be interpreted as projections rather than observed outcomes, with the 1-year estimates representing the most reliable time point for this cohort. Sixth, a total of 72 statistical tests were performed across safety outcomes. Benjamini–Hochberg FDR correction was applied to all safety comparisons, and findings that did not survive correction should be considered hypothesis-generating. Finally, although the TriNetX Global Collaborative Network spans multiple countries and healthcare settings, it is weighted toward large healthcare organizations with robust electronic health record infrastructure, which may limit generalizability to smaller community practices or resource-limited settings.

## 6. Conclusions

This propensity score-matched analysis of over 6000 patients with RRMM suggests that renal impairment, whether severe or moderate, is not associated with inferior efficacy of CAR-T cell therapy or bispecific antibody therapy, as measured by overall survival and time to next treatment over a 3-year follow-up period. However, these findings are derived from a retrospective, observational design and should be interpreted with the recognition that treated patients with RI likely represent a selected population, and that unmeasured confounders, including cytogenetic risk, disease burden, and performance status, could not be accounted for.

These findings provide a real-world evidence base supporting the feasibility of broadening treatment eligibility criteria to include patients across the GFR spectrum. They should be viewed as hypothesis-generating and as a rationale for prospective validation rather than as definitive evidence of equivalent outcomes.

From a clinical standpoint, renal impairment should inform treatment planning rather than preclude access to these agents. Grade ≥ 3 anemia was significantly elevated across all four comparisons; thrombocytopenia was significantly increased in both severe RI cohorts, and AKI risk demonstrated a severity-dependent relationship with baseline renal dysfunction. The strongest signal was observed in the BsAb severe RI group. Infections were also transiently elevated in the BsAb severe RI group during the first month, supporting heightened infection surveillance and antimicrobial prophylaxis in this subgroup during the early post-treatment period. The consistent increase in hematologic toxicity and AKI risk observed across both modalities highlights the importance of integrating renal function into pre-treatment risk stratification and supportive care protocols. Clinicians should anticipate the need for proactive transfusion support, erythropoiesis-stimulating agents, nephroprotective measures, and, in the case of CAR-T therapy, lymphodepletion regimen adaptation in patients with significant renal dysfunction. The comparable CRS and ICANS profiles across renal strata provide additional reassurance that these therapies can be administered with standard toxicity management frameworks, though vigilance for hematologic and renal complications is essential.

Prospective studies incorporating granular GFR stratification, cytogenetic risk data, and standardized IMWG response assessments are needed. Such studies would further refine risk stratification, optimize treatment selection, and establish evidence-based supportive care protocols for this common and clinically challenging patient population. Until such data are available, the present findings provide a comprehensive real-world evidence base supporting broadened access to both CAR-T therapy and bispecific antibodies for patients with RRMM and concomitant renal impairment.

## Figures and Tables

**Table 1:** Baseline Characteristics of CAR-T Cell Therapy Cohorts After Propensity Score Matching.

Characteristic		eGFR <30 vs. eGFR >60 (*n* = 281 Pairs)			eGFR 30–60 vs. eGFR >60 (*n* = 878 Pairs)		
Category	Variable	eGFR <30 (*n* = 281)	eGFR >60 (*n* = 281)	SMD	eGFR 30–60 (*n* = 878)	eGFR >60 (*n* = 878)	SMD
**Demographics**	Age, mean ± SD	64.8 ± 9.8	64.3 ± 9.9	0.020	66.5 ± 8.7	66.3 ± 8.4	0.009
	Male sex	151 (53.7%)	164 (58.4%)	0.021	483 (55.0%)	492 (56.0%)	0.020
	White race	166 (59.1%)	156 (55.5%)	0.003	660 (75.2%)	659 (75.1%)	0.003
	Black/African American	47 (16.7%)	52 (18.5%)	0.007	127 (14.5%)	129 (14.7%)	0.006
	Hispanic or Latino	10 (3.6%)	10 (3.6%)	0.014	24 (2.7%)	26 (3.0%)	0.018
**Comorbidities**	Ischemic heart disease	48 (17.1%)	43 (15.3%)	0.016	216 (24.6%)	222 (25.3%)	0.017
	Heart failure	65 (23.1%)	57 (20.3%)	0.023	175 (19.9%)	167 (19.0%)	0.023
	Obesity	40 (18.1%)	30 (13.6%)	0.036	190 (21.6%)	203 (23.1%)	0.037
	COPD	14 (5.0%)	14 (5.0%)	0.004	72 (8.2%)	73 (8.3%)	0.004
	Cerebral infarction	13 (4.6%)	13 (4.6%)	0.011	39 (4.4%)	37 (4.2%)	0.010
	Pulmonary fibrosis	10 (3.6%)	10 (3.6%)	0.006	33 (3.8%)	34 (3.9%)	0.005
**IMiDs**	Lenalidomide	100 (35.6%)	91 (32.4%)	0.048	457 (52.1%)	478 (54.4%)	0.046
	Pomalidomide	85 (30.2%)	77 (27.4%)	0.001	367 (41.8%)	367 (41.8%)	0.000
	Thalidomide	16 (5.7%)	11 (3.9%)	0.008	69 (7.9%)	71 (8.1%)	0.007
**PIs**	Bortezomib	96 (34.2%)	90 (32.0%)	0.038	297 (33.8%)	313 (35.6%)	0.038
	Carfilzomib	89 (31.7%)	85 (30.2%)	0.058	292 (33.3%)	316 (36.0%)	0.056
	Ixazomib	21 (7.5%)	11 (3.9%)	0.004	89 (10.1%)	89 (10.1%)	0.000
**Anti-CD38**	Daratumumab	104 (37.0%)	97 (34.5%)	0.039	353 (40.2%)	370 (42.1%)	0.038
	Isatuximab	10 (3.6%)	11 (3.9%)	0.054	29 (3.3%)	38 (4.3%)	0.054
**Alkylators**	Cyclophosphamide	234 (83.3%)	240 (85.4%)	0.014	699 (79.6%)	704 (80.2%)	0.015
	Melphalan	65 (29.5%)	67 (30.4%)	0.055	385 (43.9%)	409 (46.6%)	0.054
	Cisplatin	27 (9.6%)	29 (10.3%)	0.022	60 (6.8%)	65 (7.4%)	0.023
**Other**	Doxorubicin	52 (18.5%)	55 (19.6%)	0.026	120 (13.7%)	128 (14.6%)	0.025
	Etoposide	37 (13.2%)	39 (13.9%)	0.019	85 (9.7%)	90 (10.3%)	0.020
**Lines of therapy**	Line 3	131 (46.6%)	124 (44.1%)	0.055	442 (50.3%)	466 (53.1%)	0.056
	Line 4	86 (30.6%)	82 (29.2%)	0.055	308 (35.1%)	331 (37.7%)	0.054
	Line 5	53 (18.9%)	52 (18.5%)	0.053	175 (19.9%)	194 (22.1%)	0.052
**Transplant**	HPC/auto transplant	87 (31.0%)	87 (31.0%)	0.039	361 (41.1%)	378 (43.1%)	0.040
	Auto SCT	59 (21.0%)	61 (21.7%)	0.022	261 (29.7%)	270 (30.8%)	0.022

Data are presented as mean ± standard deviation for continuous variables and *n* (%) for categorical variables. Two comparisons are shown: severe renal impairment (eGFR <30 mL/min/1.73 m^2^) versus preserved renal function (eGFR >60 mL/min/1.73 m^2^), with 281 matched pairs; and moderate renal impairment (eGFR 30–60 mL/min/1.73 m^2^) versus preserved renal function, with 878 matched pairs. Propensity score matching (1:1) was performed using nearest-neighbor matching with a caliper of 0.1 pooled standard deviations. All standardized mean differences were below 0.10 after matching, confirming adequate covariate balance. SMD, standardized mean difference; eGFR, estimated glomerular filtration rate; COPD, chronic obstructive pulmonary disease; IMiDs, immunomodulatory drugs; PIs, proteasome inhibitors; HPC, hematopoietic progenitor cell; auto SCT, autologous stem cell transplantation.

**Table 2:** Baseline Characteristics of Bispecific Antibody Therapy Cohorts After Propensity Score Matching.

Characteristic		eGFR <30 vs. eGFR >60 (*n* = 645 Pairs)			eGFR 30–60 vs. eGFR >60 (*n* = 1158 Pairs)		
Category	Variable	eGFR <30 (*n* = 645)	eGFR >60 (*n* = 645)	SMD	eGFR 30–60 (*n* = 1158)	eGFR >60 (*n* = 1158)	SMD
**Demographics**	Age, mean ± SD	68.9 ± 10.0	68.7 ± 9.65	0.017	69.0 ± 9.39	69.0 ± 9.4	0.003
	Male sex	329 (51.0%)	316 (49.0%)	0.040	619 (53.5%)	621 (53.6%)	0.004
	White race	365 (56.6%)	364 (56.4%)	0.003	749 (64.7%)	756 (65.3%)	0.013
	Black/African American	176 (27.3%)	171 (26.5%)	0.018	245 (21.2%)	230 (19.9%)	0.032
	Hispanic or Latino	35 (5.4%)	34 (5.3%)	0.007	52 (4.5%)	46 (4.0%)	0.026
**Comorbidities**	Heart failure	214 (33.2%)	211 (32.7%)	0.010	255 (22.0%)	258 (22.3%)	0.006
	Ischemic heart disease	178 (27.6%)	163 (25.3%)	0.053	292 (25.2%)	281 (24.3%)	0.022
	Obesity	151 (23.4%)	160 (24.8%)	0.033	228 (19.7%)	225 (19.4%)	0.007
	COPD	69 (10.7%)	58 (9.0%)	0.057	121 (10.4%)	128 (11.1%)	0.020
	Cerebral infarction	40 (6.2%)	41 (6.4%)	0.006	63 (5.4%)	67 (5.8%)	0.015
	Pulmonary fibrosis	17 (2.6%)	19 (2.9%)	0.019	41 (3.5%)	40 (3.5%)	0.005
**IMiDs**	Lenalidomide	337 (52.2%)	351 (54.4%)	0.044	687 (59.3%)	682 (58.9%)	0.009
	Pomalidomide	274 (42.5%)	278 (43.1%)	0.013	558 (48.2%)	577 (49.8%)	0.033
	Thalidomide	32 (5.0%)	28 (4.3%)	0.030	99 (8.5%)	97 (8.4%)	0.006
**PIs**	Bortezomib	286 (44.3%)	297 (46.0%)	0.034	466 (40.2%)	481 (41.5%)	0.026
	Carfilzomib	227 (35.2%)	235 (36.4%)	0.026	394 (34.0%)	401 (34.6%)	0.013
	Ixazomib	68 (10.5%)	74 (11.5%)	0.030	146 (12.6%)	138 (11.9%)	0.021
**Anti-CD38**	Daratumumab	333 (51.6%)	342 (53.0%)	0.028	563 (48.6%)	553 (47.8%)	0.017
	Isatuximab	30 (4.7%)	31 (4.8%)	0.007	55 (4.8%)	63 (5.4%)	0.031
**Alkylators**	Cyclophosphamide	280 (43.4%)	300 (46.5%)	0.062	455 (39.3%)	450 (38.9%)	0.009
	Melphalan	147 (22.8%)	149 (23.1%)	0.007	310 (26.8%)	320 (27.6%)	0.019
	Cisplatin	27 (4.2%)	31 (4.8%)	0.030	66 (5.7%)	65 (5.6%)	0.004
**Other**	Doxorubicin	45 (7.0%)	54 (8.4%)	0.052	90 (7.8%)	83 (7.2%)	0.023
	Etoposide	44 (6.8%)	50 (7.8%)	0.036	92 (7.9%)	88 (7.6%)	0.013
**Transplant**	HPC/auto transplant	132 (20.5%)	121 (18.8%)	0.043	316 (27.3%)	315 (27.2%)	0.002
	Auto SCT	103 (16.0%)	110 (17.1%)	0.029	236 (20.4%)	232 (20.0%)	0.009
**Lines of therapy**	Line 3	255 (39.5%)	263 (40.8%)	0.025	462 (39.9%)	464 (40.1%)	0.004
	Line 4	145 (22.5%)	150 (23.3%)	0.019	275 (23.7%)	273 (23.6%)	0.004
	Line 5	71 (11.0%)	68 (10.5%)	0.015	138 (11.9%)	135 (11.7%)	0.008

Data are presented as mean ± standard deviation for continuous variables and *n* (%) for categorical variables. Two comparisons are shown: severe renal impairment (eGFR <30 mL/min/1.73 m^2^) versus preserved renal function (eGFR > 60 mL/min/1.73 m^2^), with 645 matched pairs; and moderate renal impairment (eGFR 30–60 mL/min/1.73 m^2^) versus preserved renal function, with 1158 matched pairs. Propensity score matching (1:1) was performed using nearest-neighbor matching with a caliper of 0.1 pooled standard deviations. All standardized mean differences were below 0.10 after matching, confirming adequate covariate balance. SMD, standardized mean difference; eGFR, estimated glomerular filtration rate; COPD, chronic obstructive pulmonary disease; IMiDs, immunomodulatory drugs; PIs, proteasome inhibitors; HPC, hematopoietic progenitor cell; auto SCT, autologous stem cell transplantation.

**Table 3:** Summary of Efficacy Outcomes After CAR-T Cell Therapy and Bispecific Antibody Therapy by Renal Function Stratum.

Outcome	Comparison	Modality	HR (95% CI)	*p*-Value
Overall Survival	Severe RI vs. Preserved RI	CAR-T	1.08 (0.76–1.52)	0.68
Overall Survival	Severe RI vs. Preserved RI	BsAb	1.18 (0.97–1.43)	0.11
Overall Survival	Moderate RI vs. Preserved RI	CAR-T	1.06 (0.87–1.30)	0.55
Overall Survival	Moderate RI vs. Preserved RI	BsAb	1.07 (0.92–1.25)	0.40
TTNT	Severe RI vs. Preserved RI	CAR-T	1.15 (0.87–1.51)	0.33
TTNT	Severe RI vs. Preserved RI	BsAb	1.07 (0.91–1.26)	0.40
TTNT	Moderate RI vs. Preserved RI	CAR-T	1.03 (0.89–1.19)	0.73
TTNT	Moderate RI vs. Preserved RI	BsAb	1.02 (0.91–1.14)	0.72

RI, renal impairment; HR, hazard ratio; CI, confidence interval; CAR-T, chimeric antigen receptor T-cell therapy; BsAb, bispecific antibody; TTNT, time to next treatment. Hazard ratios (HR) with 95% confidence intervals (CI) and *p*-values are shown for overall survival and time to next treatment (TTNT) across renal function strata. Each comparison was performed within propensity score-matched cohorts: severe renal impairment (eGFR <30 mL/min/1.73 m^2^) versus preserved renal function (eGFR >60 mL/min/1.73 m^2^), and moderate renal impairment (eGFR 30–60 mL/min/1.73 m^2^) versus preserved renal function. HRs > 1.0 indicate higher risk in the renal impairment group. None of the comparisons reached statistical significance (*p* < 0.05). TTNT was defined as death from any cause or initiation of subsequent myeloma-directed therapy, whichever occurred first.

**Table 4:** Safety Outcomes After CAR-T and Bispecific Antibody Therapy by Renal Function Stratum—Risk Ratios at 1, 3, and 6 Months.

Adverse Outcome	Time Point	CAR-T eGFR <30 vs. >60 (*n* = 281)	CAR-T eGFR 30–60 vs. >60 (*n* = 878)	Bispecific eGFR <30 vs. >60 (*n* = 645)	Bispecific eGFR 30–60 vs. >60 (*n* = 1158)
		**RR (95% CI)/*p*-Value**	**RR (95% CI)/*p*-Value**	**RR (95% CI)/*p*-Value**	**RR (95% CI)/*p*-Value**
CRS	1 Month	0.97 (0.76–1.23) *p* = 0.790	1.01 (0.90–1.13) *p* = 0.923	0.87 (0.69–1.09) *p* = 0.222	0.89 (0.75–1.04) *p* = 0.144
ICANS	1 Month	0.82 (0.45–1.49) *p* = 0.510	1.17 (0.86–1.59) *p* = 0.309	1.10 (0.61–2.00) *p* = 0.754	1.00 (0.61–1.63) *p* = 1.000
AKI†	1 Month	1.43 (0.97–2.11) *p* = 0.070	**1.38 (1.11–1.71) *p* = 0.003**	**2.50 (2.00–3.12) *p* < 0.001**	**1.70 (1.41–2.06) *p* < 0.001**
Grade ≥ 3 Anemia	1 Month	**1.32 (1.14–1.52) *p* < 0.001**	**1.17 (1.05–1.31) *p* = 0.005**	**1.46 (1.29–1.65) *p* < 0.001**	**1.12 (1.00–1.25) *p* = 0.046**
	3 Months	**1.27 (1.11–1.46) *p* < 0.001**	**1.16 (1.04–1.29) *p* = 0.005**	**1.37 (1.22–1.53) *p* < 0.001**	**1.12 (1.01–1.23) *p* = 0.031**
	6 Months	**1.26 (1.11–1.43) *p* < 0.001**	**1.15 (1.04–1.26) *p* = 0.007**	**1.37 (1.23–1.52) *p* < 0.001**	**1.11 (1.01–1.22) *p* = 0.026**
Grade ≥ 3 Thrombocytopenia	1 Month	**1.17 (1.03–1.35) *p* = 0.020**	1.06 (0.96–1.16) *p* = 0.272	**1.26 (1.06–1.50) *p* = 0.009**	1.14 (0.98–1.32) *p* = 0.094
	3 Months	**1.21 (1.06–1.38) *p* = 0.004**	1.05 (0.95–1.15) *p* = 0.340	**1.21 (1.03–1.41) *p* = 0.018**	1.11 (0.97–1.27) *p* = 0.124
	6 Months	**1.20 (1.06–1.37) *p* = 0.004**	1.04 (0.96–1.14) *p* = 0.339	**1.23 (1.06–1.43) *p* = 0.006**	1.09 (0.96–1.24) *p* = 0.171
Grade ≥ 3 Neutropenia	1 Month	0.97 (0.91–1.03) *p* = 0.310	1.03 (0.99–1.07) *p* = 0.102	1.08 (0.88–1.34) *p* = 0.455	1.01 (0.87–1.18) *p* = 0.879
	3 Months	0.96 (0.90–1.01) *p* = 0.140	1.02 (0.99–1.06) *p* = 0.148	1.02 (0.87–1.19) *p* = 0.813	0.98 (0.87–1.10) *p* = 0.757
	6 Months	0.96 (0.91–1.02) *p* = 0.170	1.02 (0.99–1.05) *p* = 0.162	1.04 (0.91–1.18) *p* = 0.572	0.99 (0.89–1.10) *p* = 0.799
Infections (all grade)	1 Month	1.17 (0.84–1.63) *p* = 0.340	1.03 (0.86–1.23) *p* = 0.729	**1.29 (1.06–1.58) *p* = 0.011**	1.06 (0.91–1.25) *p* = 0.464
	3 Months	1.25 (0.97–1.60) *p* = 0.080	1.03 (0.89–1.19) *p* = 0.678	1.09 (0.94–1.26) *p* = 0.269	0.98 (0.87–1.11) *p* = 0.788
	6 Months	1.22 (0.98–1.52) *p* = 0.080	1.04 (0.92–1.18) *p* = 0.551	1.08 (0.97–1.22) *p* = 0.260	0.99 (0.89–1.10) *p* = 0.830
Hypogammaglobulinemia	1 Month	1.04 (0.80–1.36) *p* = 0.780	0.95 (0.82–1.10) *p* = 0.462	1.16 (0.97–1.40) *p* = 0.111	1.04 (0.90–1.20) *p* = 0.595
	3 Months	1.14 (0.94–1.39) *p* = 0.170	0.99 (0.89–1.10) *p* = 0.885	1.03 (0.89–1.18) *p* = 0.729	1.02 (0.91–1.14) *p* = 0.728
	6 Months	**1.19 (1.01–1.41) *p* = 0.040**	0.99 (0.90–1.08) *p* = 0.775	1.02 (0.89–1.16) *p* = 0.821	1.01 (0.91–1.11) *p* = 0.890

Data are presented as risk ratios (95% confidence intervals); *p*-values. Statistically significant comparisons (*p* < 0.05) are shown in bold. CRS, ICANS, and AKI were assessed at 1 month only; all other outcomes were assessed at 1, 3, and 6 months. Grade ≥ 3 cytopenias were defined as hemoglobin < 8.0 g/dL (anemia), platelets ≤ 50 × 10^3^/µL (thrombocytopenia), and absolute neutrophil count < 1.0 × 10^3^/µL (neutropenia). Infection data represent all-grade events. Each comparison was performed within propensity score-matched cohorts: severe renal impairment (eGFR <30 mL/min/1.73 m^2^) versus preserved renal function (eGFR > 60 mL/min/1.73 m^2^), and moderate renal impairment (eGFR 30–60 mL/min/1.73 m^2^) versus preserved renal function. *n* refers to matched pairs per group. RR, risk ratio; CI, confidence interval; CRS, cytokine release syndrome; ICANS, immune effector cell-associated neurotoxicity syndrome; AKI, acute kidney injury; eGFR, estimated glomerular filtration rate; CAR-T, chimeric antigen receptor T-cell; BsAb, bispecific antibody.

## Data Availability

The data used in this study were obtained from the TriNetX Global Collaborative Network (https://trinetx.com). Access is subject to institutional agreements with TriNetX. Aggregate-level results are available from the corresponding author upon reasonable request.

## Supplementary Materials

The following supporting information can be downloaded at: https://www.mdpi.com/article/10.3390/cancers18142311/s1. Supplementary Figure S1: Safety Outcomes After CAR-T Cell Therapy in Patients With eGFR <30 vs. eGFR >60 mL/min/1.73 m^2^ (*n* = 281 Propensity-Matched Pairs). Forest plots depicting cumulative event rates and risk ratios (RR) with 95% confidence intervals (CI) for safety outcomes at 1, 3, and 6 months after CAR-T cell therapy, comparing propensity-matched patients with baseline eGFR <30 vs. >60 mL/min/1.73 m^2^. Safety outcomes include cytokine release syndrome (CRS; 1 month only), immune effector cell-associated neurotoxicity syndrome (ICANS; 1 month only), acute kidney injury (AKI; 1 month only), grade ≥ 3 anemia, grade ≥ 3 thrombocytopenia, grade ≥ 3 neutropenia, infections, and hypogammaglobulinemia. Diamond markers denote statistically significant comparisons (*p* < 0.05); circle markers denote non-significant comparisons. Supplementary Figure S2: Safety Outcomes After CAR-T Cell Therapy in Patients With eGFR 30–60 vs. eGFR >60 mL/min/1.73 m^2^ (*n* = 878 Propensity-Matched Pairs). Forest plots depicting cumulative event rates and risk ratios (RR) with 95% confidence intervals (CI) for safety outcomes at 1, 3, and 6 months after CAR-T cell therapy, comparing propensity-matched patients with baseline eGFR 30–60 vs. >60 mL/min/1.73 m^2^. Safety outcomes include CRS (1 month only), ICANS (1 month only), AKI (1 month only), grade ≥ 3 anemia, grade ≥ 3 thrombocytopenia, grade ≥ 3 neutropenia, infections, and hypogammaglobulinemia. Diamond markers denote statistically significant comparisons (*p* < 0.05); circle markers denote non-significant comparisons. Supplementary Figure S3: Safety Outcomes After Bispecific Antibody Therapy in Patients With eGFR <30 vs. eGFR >60 mL/min/1.73 m^2^ (*n* = 645 Propensity-Matched Pairs). Forest plots depicting cumulative event rates and risk ratios (RR) with 95% confidence intervals (CI) for safety outcomes at 1, 3, and 6 months after bispecific antibody therapy, comparing propensity-matched patients with baseline eGFR <30 vs. >60 mL/min/1.73 m^2^. Safety outcomes include CRS (1 month only), ICANS (1 month only), AKI (1 month only), grade ≥ 3 anemia, grade ≥ 3 thrombocytopenia, grade ≥ 3 neutropenia, infections, and hypogammaglobulinemia. Diamond markers denote statistically significant comparisons (*p* < 0.05); circle markers denote non-significant comparisons. Supplementary Figure S4: Safety Outcomes After Bispecific Antibody Therapy in Patients With eGFR 30–60 vs. eGFR > 60 mL/min/1.73 m^2^ (*n* = 1158 Propensity-Matched Pairs). Forest plots depicting cumulative event rates and risk ratios (RR) with 95% confidence intervals (CI) for safety outcomes at 1, 3, and 6 months after bispecific antibody therapy, comparing propensity-matched patients with baseline eGFR 30–60 vs. >60 mL/min/1.73 m^2^. Safety outcomes include CRS (1 month only), ICANS (1 month only), AKI (1 month only), grade ≥ 3 anemia, grade ≥ 3 thrombocytopenia, grade ≥ 3 neutropenia, infections, and hypogammaglobulinemia. Diamond markers denote statistically significant comparisons (*p* < 0.05); circle markers denote non-significant comparisons. Supplementary Table S1: Diagnosis, Procedure, Medication, and Laboratory Codes Used in This Study. Comprehensive listing of all International Classification of Diseases, Tenth Revision, Clinical Modification (ICD-10-CM) diagnosis codes, International Classification of Diseases, Tenth Revision, Procedure Coding System (ICD-10-PCS) procedure codes, RxNorm medication codes used for cohort identification, exposure definitions, renal function stratification, propensity score matching covariates, and outcome ascertainment in this study. Supplementary Table S2: Cumulative Mortality and Time to Next Treatment After CAR-T Cell Therapy at Fixed Timepoints (1, 2, and 3 Years), Stratified by Baseline eGFR. Supplementary Table S3: Cumulative Mortality and Time to Next Treatment After Bispecific Antibody Therapy at Fixed Timepoints (1, 2, and 3 Years), Stratified by Baseline eGFR. Supplementary Table S4: Product-Stratified 1-Year Mortality and Time-to-Next-Treatment Outcomes After CAR-T Cell Therapy by Renal-Function Stratum. Hazard ratios (95% CI) and log-rank *p*-values for 1-year mortality and TTNT, stratified by individual CAR-T product (idecabtagene vicleucel, ciltacabtagene autoleucel). Comparisons were performed within each product between patients with eGFR <30 or eGFR 30–60 mL/min/1.73 m^2^ and those with eGFR >60 mL/min/1.73 m^2^. Supplementary Table S5: Product-Stratified 1-Year Mortality and Time-to-Next-Treatment Outcomes After Bispecific Antibody Therapy by Renal-Function Stratum. Hazard ratios (95% CI) and log-rank *p*-values for 1-year mortality and TTNT, stratified by individual bispecific antibody agent (teclistamab, talquetamab, elranatamab). Comparisons were performed within each agent between patients with eGFR <30 or eGFR 30–60 mL/min/1.73 m^2^ and those with eGFR >60 mL/min/1.73 m^2^. Supplementary Table S6: Product-Stratified Safety Outcomes After CAR-T Cell Therapy by Renal-Function Stratum. Risk ratios with 95% confidence intervals for safety outcomes at 1, 3, and 6 months, stratified by individual CAR-T product (idecabtagene vicleucel, ciltacabtagene autoleucel). Comparisons were performed within each product between patients with eGFR <30 or eGFR 30–60 mL/min/1.73 m^2^ and those with eGFR >60 mL/min/1.73 m^2^. Supplementary Table S7: Product-Stratified Safety Outcomes After Bispecific Antibody Therapy by Renal-Function Stratum. Risk ratios with 95% confidence intervals for safety outcomes at 1, 3, and 6 months, stratified by individual bispecific antibody agent (teclistamab, talquetamab, elranatamab). Comparisons were performed within each agent between patients with eGFR <30 or eGFR 30–60 mL/min/1.73 m^2^ and those with eGFR >60 mL/min/1.73 m^2^. Supplementary Table S8: Benjamini–Hochberg False Discovery Rate (FDR)–Adjusted Safety Outcome *p*-Values. Raw *p*-values and FDR-adjusted q-values for all 72 pairwise safety comparisons reported in Table 4, encompassing CRS, ICANS, AKI, grade ≥ 3 anemia, grade ≥ 3 thrombocytopenia, grade ≥ 3 neutropenia, infections, and hypogammaglobulinemia at 1, 3, and 6 months across all four renal-function comparisons, adjusted using the Benjamini–Hochberg procedure to control the false discovery rate at 5%.

## Abbreviations

The following abbreviations are used in this manuscript:

AKI	Acute kidney injury
BsAb	Bispecific antibody
BCMA	B-cell maturation antigen
CAR-T	Chimeric antigen receptor T-cell
CI	Confidence interval
CKD	Chronic kidney disease
cilta-cel	Ciltacabtagene autoleucel
COPD	Chronic obstructive pulmonary disease
CPT	Current Procedural Terminology
CRS	Cytokine release syndrome
CrCl	Creatinine clearance
EHR	Electronic health record
eGFR	Estimated glomerular filtration rate
ESRD	End-stage renal disease
GPRC5D	G protein-coupled receptor class C group 5 member D
HCPCS	Healthcare Common Procedure Coding System
HIPAA	Health Insurance Portability and Accountability Act
HR	Hazard ratio
ICD-10-CM	International Classification of Diseases, Tenth Revision, Clinical Modification
ICD-10-PCS	International Classification of Diseases, Tenth Revision, Procedure Coding System
ICANS	Immune effector cell-associated neurotoxicity syndrome
ide-cel	Idecabtagene vicleucel
IMiDs	Immunomodulatory drugs
IMWG	International Myeloma Working Group
ORR	Overall response rate
OS	Overall survival
PFS	Progression-free survival
PIs	Proteasome inhibitors
PSM	Propensity score matching
RI	Renal impairment
RR	Risk ratio
RRMM	Relapsed/refractory multiple myeloma
SMD	Standardized mean difference
TTNT	Time to next treatment

## References

[1] Berdeja J.G., Madduri D., Usmani S.Z., Jakubowiak A., Agha M., Cohen A.D., Stewart A.K., Hari P., Htut M., Lesokhin A. (2021). Ciltacabtagene Autoleucel, a B-Cell Maturation Antigen-Directed Chimeric Antigen Receptor T-Cell Therapy in Patients with Relapsed or Refractory Multiple Myeloma (CARTITUDE-1): A Phase 1b/2 Open-Label Study. Lancet.

[2] Rodriguez-Otero P., Ailawadhi S., Arnulf B., Patel K., Cavo M., Nooka A.K., Manier S., Callander N., Costa L.J., Vij R. (2023). Ide-Cel or Standard Regimens in Relapsed and Refractory Multiple Myeloma. N. Engl. J. Med..

[3] Moreau P., Garfall A.L., Van De Donk N.W.C.J., Nahi H., San-Miguel J.F., Oriol A., Nooka A.K., Martin T., Rosinol L., Chari A. (2022). Teclistamab in Relapsed or Refractory Multiple Myeloma. N. Engl. J. Med..

[4] Lesokhin A.M., Tomasson M.H., Arnulf B., Bahlis N.J., Miles Prince H., Niesvizky R., Rodrίguez-Otero P., Martinez-Lopez J., Koehne G., Touzeau C. (2023). Elranatamab in Relapsed or Refractory Multiple Myeloma: Phase 2 MagnetisMM-3 Trial Results. Nat. Med..

[5] Chari A., Minnema M.C., Berdeja J.G., Oriol A., Van De Donk N.W.C.J., Rodríguez-Otero P., Askari E., Mateos M.-V., Costa L.J., Caers J. (2022). Talquetamab, a T-Cell–Redirecting GPRC5D Bispecific Antibody for Multiple Myeloma. N. Engl. J. Med..

[6] Dimopoulos M.A., Merlini G., Bridoux F., Leung N., Mikhael J., Harrison S.J., Kastritis E., Garderet L., Gozzetti A., Van De Donk N.W.C.J. (2023). Management of Multiple Myeloma-Related Renal Impairment: Recommendations from the International Myeloma Working Group. Lancet Oncol..

[7] Van De Donk N.W.C.J., Moreau P., San-Miguel J.F., Mateos M.-V., Dimopoulos M.A., Zweegman S., Gay F., Engelhardt M., Mina R., Zamagni E. (2025). Optimising T-Cell Immunotherapy in Patients with Multiple Myeloma: Practical Considerations from the European Myeloma Network. Lancet Haematol..

[8] Bridoux F., Leung N., Belmouaz M., Royal V., Ronco P., Nasr S.H., Fermand J.P. (2021). Management of Acute Kidney Injury in Symptomatic Multiple Myeloma. Kidney Int..

[9] Wang L., Liu C., Song H., Yuan J., Zha Y., Deng Y. (2024). Update on Kidney Injury Caused by Multiple Myeloma. Ann. Hematol..

[10] Rosner M.H., Perazella M.A. (2017). Acute Kidney Injury in Patients with Cancer. N. Engl. J. Med..

[11] Sidana S., Peres L.C., Hashmi H., Hosoya H., Ferreri C., Khouri J., Dima D., Atrash S., Voorhees P., Simmons G. (2023). Idecabtagene Vicleucel Chimeric Antigen Receptor T-Cell Therapy for Relapsed/Refractory Multiple Myeloma with Renal Impairment. Haematologica.

[12] Grieb N., Wiemers T., Born P., Fandrei D., Fischer L., Ferle M., Franke S., Keller J., Wang S.Y., Jentzsch M. (2026). Impact of Renal Impairment and Lymphodepletion Regimen on Outcomes after CAR T Cell Therapy in Relapsed/Refractory Multiple Myeloma. Transplant. Cell. Ther..

[13] Filippatos C., Ntanasis-Stathopoulos I., Briasoulis A., Malandrakis P., Terpos E., Gavriatopoulou M. (2026). Real-World Experience with Approved CAR T-Cell Therapies Ciltacabtagene Autoleucel and Idecabtagene Vicleucel in 1272 Relapsed/Refractory Multiple Myeloma Patients. Cancers.

[14] Mahadevia H., Modi K., Arya Y., Syal A., El-Husseiny K.M., Modi S., Bansal V., Vodnala D., Khumri T., Cossor F. (2026). Impact of Chronic Kidney Disease on Toxicity Patterns and Economic Burden in CAR T-Cell Therapy for Multiple Myeloma: A National Analysis. J. Clin. Oncol..

[15] Ntanasis-Stathopoulos I., Manganas S., Filippatos C., Karamouzis K., Gavriatopoulou M., Kastritis E., Terpos E., Dimopoulos M. (2026). The Role of Bispecific Antibodies in Relapsed/Refractory Multiple Myeloma with Renal Impairment: A Systematic Review. Am. J. Hematol..

[16] Dima D., Afrough A., Goel U., Grajales-Cruz A.F., Khouri J., Julian K., Pasvolsky O., Banerjee R., Razzo B., Ferreri C.J. (2025). Teclistamab for Patients with Heavily Pretreated Relapsed/Refractory Multiple Myeloma and Renal Impairment. Blood Adv..

[17] Jiang H., Ahlawat Y., Steinberg A. (2025). Using Bispecific Antibodies in a Patient with Peritoneal Dialysis for Relapsed Multiple Myeloma. J. Oncol. Pharm. Pract..

[18] Ogiya D., Suzuki R., Goto S., Nakagawa Y., Nasukawa M., Iwata S., Kawai H., Toyosaki M., Machida S., Onizuka M. (2025). Efficacy and Safety of Bispecific Antibodies in Multiple Myeloma Patients with End-Stage Renal Disease Undergoing Hemodialysis: A Case Report of Elranatamab and a Literature Review. Ann. Hematol..

[19] Hoffmann M., Jeker B., Huynh-Do U., Banz Y., Godau J., Weber E., Bacher U., Pabst T. (2025). Elranatamab for Relapsed/Refractory Multiple Myeloma with Severe Renal Impairment Requiring Hemodialysis. Hematol. Oncol..

[20] Chen Z., Chen Y., Liu J., Sun Y., Zhang X., Shao L., Wang D., Wang X., Chen W., Sang W. (2025). Nephrotoxicity of CAR-T Therapy in Patients with Relapsed and Refractory Multiple Myeloma. Int. Urol. Nephrol..

[21] Charkviani M., Brochero M.J.V., Mohan A., Vaughan L.E., Sandahl T.B., Corraes A.D.M.S., Lin Y., Leung N., Herrmann S.M. (2025). Incidence of Acute Kidney Injury in Relapsed and Refractory Multiple Myeloma Treated with Teclistamab versus CAR-T Cells. Nephrol. Dial. Transplant..

[22] Wu L., Feng Y., Huang Y., Feng J., Hu Y., Huang H. (2024). CAR-T Cell Therapy: Advances in Kidney-Related Diseases. Kidney Dis..

[23] Song Z., Tsou S., Martin F., Kayumov M., Xiao Y., Zhou H., Abdi R., Tullius S.G. (2026). Kidney Disease as a Driver of Immunosenescence: Mechanisms and Potential Interventions. J. Am. Soc. Nephrol..

[24] Rocchetti M.T., Cosola C., Ranieri E., Gesualdo L., Gigante M., Ranieri E. (2021). Protein-Bound Uremic Toxins and Immunity. Cytotoxic T-Cells.

[25] Reynolds G., Cliff E.R.S., Mohyuddin G.R., Popat R., Midha S., Liet Hing M.N., Harrison S.J., Kesselheim A.S., Teh B.W. (2023). Infections Following Bispecific Antibodies in Myeloma: A Systematic Review and Meta-Analysis. Blood Adv..

[26] Cordas Dos Santos D.M., Tix T., Shouval R., Gafter-Gvili A., Alberge J.-B., Cliff E.R.S., Theurich S., Von Bergwelt-Baildon M., Ghobrial I.M., Subklewe M. (2024). A Systematic Review and Meta-Analysis of Nonrelapse Mortality after CAR T Cell Therapy. Nat. Med..

[27] Mohan M., Chakraborty R., Bal S., Nellore A., Baljevic M., D’Souza A., Pappas P.G., Berdeja J.G., Callander N., Costa L.J. (2023). Recommendations on Prevention of Infections during Chimeric Antigen Receptor T-cell and Bispecific Antibody Therapy in Multiple Myeloma. Br. J. Haematol..

[28] Raje N., Anderson K., Einsele H., Efebera Y., Gay F., Hammond S.P., Lesokhin A.M., Lonial S., Ludwig H., Moreau P. (2023). Monitoring, Prophylaxis, and Treatment of Infections in Patients with MM Receiving Bispecific Antibody Therapy: Consensus Recommendations from an Expert Panel. Blood Cancer J..

[29] Ludwig H., Terpos E., Van De Donk N., Mateos M.-V., Moreau P., Dimopoulos M.-A., Delforge M., Rodriguez-Otero P., San-Miguel J., Yong K. (2023). Prevention and Management of Adverse Events during Treatment with Bispecific Antibodies and CAR T Cells in Multiple Myeloma: A Consensus Report of the European Myeloma Network. Lancet Oncol..

[30] Bhatt P., Kloock C., Comenzo R. (2023). Relapsed/Refractory Multiple Myeloma: A Review of Available Therapies and Clinical Scenarios Encountered in Myeloma Relapse. Curr. Oncol..

