# Supplementary material for: Efficacy and Safety of CAR-T Cell Therapy and Bispecific Antibodies in Relapsed/Refractory Multiple Myeloma with Renal Impairment: A Propensity Score-Matched Analysis
Source: Cancers (Basel). 2026 Jul 17;18(14):2311. doi: 10.3390/cancers18142311 (PMC13406253; doi:10.3390/cancers18142311)
Supplement: Supplementary file 1 [file cancers-18-02311-s001.zip › Supplementary_Table_S1.pdf]

Supplementary Table S1. Diagnosis, Procedure, Medication, and Laboratory Codes Used in This Study.

Coding System	Code	Description
Multiple Myeloma Cohort Identification		
ICD-10-CM	**C90.0**	Multiple myeloma
ICD-10-CM	**C90.00**	Multiple myeloma not having achieved remission
ICD-10-CM	**C90.02**	Multiple myeloma in relapse
CAR-T Cell Therapy — Exposure Codes		
RxNorm	**2536430**	Idecabtagene vicleucel
RxNorm	**2594775**	Ciltacabtagene autoleucel
ICD-10-PCS	**XW033A7**	Introduction of ciltacabtagene autoleucel into peripheral vein, percutaneous approach, new technology group 7
ICD-10-PCS	**XW043A7**	Introduction of ciltacabtagene autoleucel into central vein, percutaneous approach, new technology group 7
ICD-10-PCS	**XW033K7**	Introduction of idecabtagene vicleucel immunotherapy into peripheral vein, percutaneous approach, new technology group 7
ICD-10-PCS	**XW043K7**	Introduction of idecabtagene vicleucel immunotherapy into central vein, percutaneous approach, new technology group 7
Bispecific Antibody Therapy — Exposure Codes		
RxNorm	**2619426**	Teclistamab
RxNorm	**2644447**	Talquetamab
RxNorm	**2644880**	Elranatamab
ICD-10-PCS	**XW01348**	Introduction of teclistamab antineoplastic into subcutaneous tissue, percutaneous approach, new technology group 8
ICD-10-PCS	**XW01329**	Introduction of talquetamab antineoplastic into subcutaneous tissue, percutaneous approach, new technology group 9
ICD-10-PCS	**XW013L9**	Introduction of elranatamab antineoplastic into subcutaneous tissue, percutaneous approach, new technology group 9
Renal Function / Chronic Kidney Disease Classification		
ICD-10-CM	**N18.3**	Chronic kidney disease, stage 3 (moderate)
ICD-10-CM	**N18.4**	Chronic kidney disease, stage 4 (severe)
ICD-10-CM	**N18.5**	Chronic kidney disease, stage 5
ICD-10-CM	**N18.6**	End stage renal disease
ICD-10-CM	**Z99.2**	Dependence on renal dialysis
ICD-10-CM	**I12.0**	Hypertensive chronic kidney disease with stage 5 chronic kidney disease or end stage renal disease

Coding System	Code	Description
Safety Outcome Definitions		
ICD-10-CM	**D89.83**	Cytokine release syndrome
ICD-10-CM	**G92.00**	Immune effector cell-associated neurotoxicity syndrome, grade unspecified
ICD-10-CM	**N17**	Acute kidney failure
ICD-10-CM	**N17.0**	Acute kidney failure with tubular necrosis
ICD-10-CM	**N17.1**	Acute kidney failure with acute cortical necrosis
ICD-10-CM	**N17.2**	Acute kidney failure with medullary necrosis
ICD-10-CM	**N17.8**	Other acute kidney failure
ICD-10-CM	**N17.9**	Acute kidney failure, unspecified
ICD-10-CM	**A00–B99**	Certain infectious and parasitic diseases
ICD-10-CM	**D80.1**	Nonfamilial hypogammaglobulinemia
TNX Curated	**9014**	Hemoglobin [Mass/volume] in Blood, ≤8 g/dL (most recent value)
TNX Curated	**9020**	Platelets [#/volume] in Blood, ≤50 $\times 10^3/\mu L$ (most recent value)
TNX Curated	**9018**	Neutrophils [#/volume] in Blood, ≤1 $\times 10^3/\mu L$ (most recent value)
Propensity Score Matching Covariates (Comorbidities)		
ICD-10-CM	**I25.x**	Chronic ischemic heart disease
ICD-10-CM	**I50.x**	Heart failure
ICD-10-CM	**E66.x**	Overweight and obesity
ICD-10-CM	**I63.x**	Cerebral infarction
ICD-10-CM	**J44.x**	Chronic obstructive pulmonary disease
ICD-10-CM	**J84.1x**	Pulmonary fibrosis (interstitial pulmonary diseases with fibrosis)

Abbreviations: ICD-10-CM, International Classification of Diseases, Tenth Revision, Clinical Modification; ICD-10-PCS, International Classification of Diseases, Tenth Revision, Procedure Coding System; CRS, cytokine release syndrome; ICANS, immune effector cell-associated neurotoxicity syndrome; AKI, acute kidney injury; PSM, propensity score matching; CAR-T, chimeric antigen receptor T-cell; ide-cel, idecabtagene vicleucel; cilta-cel, ciltacabtagene autoleucel; TNX, TriNetX.
