# Supplementary material for: Efficacy and Safety of CAR-T Cell Therapy and Bispecific Antibodies in Relapsed/Refractory Multiple Myeloma with Renal Impairment: A Propensity Score-Matched Analysis
Source: Cancers (Basel). 2026 Jul 17;18(14):2311. doi: 10.3390/cancers18142311 (PMC13406253; doi:10.3390/cancers18142311)
Supplement: Supplementary file 1 [file cancers-18-02311-s001.zip › Supplementary_Table_S2.pdf]

Supplementary Table S2. Cumulative Mortality and Time to Next Treatment After CAR-T Cell Therapy at Fixed Timepoints (1, 2, and 3 Years), Stratified by Baseline eGFR.

Comparison 1: eGFR <30 vs. eGFR >60 (n=281 matched pairs)						
**Overall Survival (Mortality)**						
**Time Point**	**eGFR <30 Deaths/N (%)**	**eGFR >60 Deaths/N (%)**	**Risk Ratio (95% CI)**	**p-value**	**Hazard Ratio (95% CI)**	**Log-rank p**
1 Year	45/281 (16.0%)	47/281 (16.7%)	0.96 (0.66–1.39)	0.820	0.95 (0.63–1.43)	0.795
2 Years	63/281 (22.4%)	56/281 (19.9%)	1.13 (0.82–1.55)	0.470	1.12 (0.78–1.61)	0.529
3 Years	68/281 (24.2%)	63/281 (22.4%)	1.08 (0.80–1.46)	0.618	1.08 (0.76–1.52)	0.679
**Time to Next Treatment (TTNT)**						
**Time Point**	**eGFR <30 Events/N (%)**	**eGFR >60 Events/N (%)**	**Risk Ratio (95% CI)**	**p-value**	**Hazard Ratio (95% CI)**	**Log-rank p**
1 Year	79/281 (28.1%)	75/281 (26.7%)	1.05 (0.81–1.38)	0.705	1.07 (0.78–1.46)	0.682
2 Years	103/281 (36.7%)	91/281 (32.4%)	1.13 (0.90–1.42)	0.287	1.14 (0.86–1.52)	0.350
3 Years	108/281 (38.3%)	95/281 (33.8%)	1.14 (0.91–1.42)	0.254	1.15 (0.87–1.51)	0.349

Comparison 2: eGFR 30–60 vs. eGFR >60 (n=878 matched pairs)						
**Overall Survival (Mortality)**						
**Time Point**	**eGFR 30–60 Deaths/N (%)**	**eGFR >60 Deaths/N (%)**	**Risk Ratio (95% CI)**	**p-value**	**Hazard Ratio (95% CI)**	**Log-rank p**
1 Year	124/878 (14.1%)	115/878 (13.1%)	1.08 (0.85–1.37)	0.531	1.07 (0.83–1.38)	0.592
2 Years	176/878 (20.0%)	160/878 (18.2%)	1.10 (0.91–1.33)	0.332	1.10 (0.89–1.36)	0.398
3 Years	197/878 (22.4%)	185/878 (21.1%)	1.07 (0.89–1.27)	0.488	1.06 (0.87–1.30)	0.554
**Time to Next Treatment (TTNT)**						
**Time Point**	**eGFR 30–60 Events/N (%)**	**eGFR >60 Events/N (%)**	**Risk Ratio (95% CI)**	**p-value**	**Hazard Ratio (95% CI)**	**Log-rank p**
1 Year	242/878 (27.6%)	232/878 (26.4%)	1.04 (0.89–1.22)	0.591	1.04 (0.87–1.25)	0.647
2 Years	333/878 (37.9%)	320/878 (36.4%)	1.04 (0.92–1.18)	0.521	1.04 (0.89–1.21)	0.635
3 Years	354/878 (40.3%)	344/878 (39.2%)	1.03 (0.92–1.16)	0.626	1.03 (0.89–1.19)	0.738

OS events = all-cause mortality. TTNT events = death or initiation of next myeloma-directed therapy (whichever first). RR, risk ratio; HR, hazard ratio; CI, confidence interval. All comparisons non-significant (p>0.050) unless otherwise noted. HR derived from Cox proportional hazards model. Propensity-matched cohorts.
