# Supplementary material for: Efficacy and Safety of CAR-T Cell Therapy and Bispecific Antibodies in Relapsed/Refractory Multiple Myeloma with Renal Impairment: A Propensity Score-Matched Analysis
Source: Cancers (Basel). 2026 Jul 17;18(14):2311. doi: 10.3390/cancers18142311 (PMC13406253; doi:10.3390/cancers18142311)
Supplement: Supplementary file 1 [file cancers-18-02311-s001.zip › Supplementary_Table_S3.pdf]

Supplementary Table S3. Cumulative Mortality and Time to Next Treatment After Bispecific Antibody Therapy at Fixed Timepoints (1, 2, and 3 Years), Stratified by Baseline eGFR.

Comparison 1: eGFR <30 vs. eGFR >60 (n=645 matched pairs)						
**Overall Survival (Mortality)**						
**Time Point**	**eGFR <30 Deaths/N (%)**	**eGFR >60 Deaths/N (%)**	**Risk Ratio (95% CI)**	**p-value**	**Hazard Ratio (95% CI)**	**Log-rank p**
1 Year	176/645 (27.3%)	148/645 (22.9%)	1.19 (0.98–1.44)	0.072	1.23 (0.99–1.53)	0.061
2 Years	205/645 (31.7%)	180/645 (27.9%)	1.14 (0.96–1.35)	0.128	1.18 (0.97–1.45)	0.100
3 Years	209/645 (32.4%)	184/645 (28.5%)	1.14 (0.96–1.34)	0.131	1.18 (0.97–1.43)	0.108
**Time to Next Treatment (TTNT)**						
**Time Point**	**eGFR <30 Events/N (%)**	**eGFR >60 Events/N (%)**	**Risk Ratio (95% CI)**	**p-value**	**Hazard Ratio (95% CI)**	**Log-rank p**
1 Year	279/645 (43.3%)	259/645 (40.2%)	1.08 (0.95–1.23)	0.259	1.13 (0.96–1.34)	0.154
2 Years	304/645 (47.1%)	298/645 (46.2%)	1.02 (0.91–1.15)	0.738	1.07 (0.92–1.26)	0.380
3 Years	308/645 (47.8%)	302/645 (46.8%)	1.02 (0.91–1.14)	0.738	1.07 (0.91–1.26)	0.400

Comparison 2: eGFR 30–60 vs. eGFR >60 (n=1,158 matched pairs)						
**Overall Survival (Mortality)**						
**Time Point**	**eGFR 30–60 Deaths/N (%)**	**eGFR >60 Deaths/N (%)**	**Risk Ratio (95% CI)**	**p-value**	**Hazard Ratio (95% CI)**	**Log-rank p**
1 Year	270/1,158 (23.3%)	249/1,158 (21.5%)	1.08 (0.93–1.26)	0.295	1.11 (0.94–1.32)	0.228
2 Years	319/1,158 (27.5%)	302/1,158 (26.1%)	1.06 (0.92–1.21)	0.425	1.09 (0.93–1.27)	0.304
3 Years	323/1,158 (27.9%)	310/1,158 (26.8%)	1.04 (0.91–1.19)	0.544	1.07 (0.92–1.25)	0.399
**Time to Next Treatment (TTNT)**						
**Time Point**	**eGFR 30–60 Events/N (%)**	**eGFR >60 Events/N (%)**	**Risk Ratio (95% CI)**	**p-value**	**Hazard Ratio (95% CI)**	**Log-rank p**
1 Year	530/1,158 (45.8%)	530/1,158 (45.8%)	1.00 (0.92–1.09)	1.000	1.04 (0.92–1.17)	0.580
2 Years	596/1,158 (51.5%)	596/1,158 (51.5%)	1.00 (0.92–1.08)	1.000	1.03 (0.92–1.16)	0.599
3 Years	601/1,158 (51.9%)	606/1,158 (52.3%)	0.99 (0.92–1.07)	0.835	1.02 (0.91–1.14)	0.719

OS events = all-cause mortality. TTNT events = death or initiation of next myeloma-directed therapy (whichever first). RR, risk ratio; HR, hazard ratio; CI, confidence interval. All comparisons non-significant (p>0.050) unless otherwise noted. HR derived from Cox proportional hazards model. Propensity-matched cohorts. Source:
