# Supplementary material for: Efficacy and Safety of CAR-T Cell Therapy and Bispecific Antibodies in Relapsed/Refractory Multiple Myeloma with Renal Impairment: A Propensity Score-Matched Analysis
Source: Cancers (Basel). 2026 Jul 17;18(14):2311. doi: 10.3390/cancers18142311 (PMC13406253; doi:10.3390/cancers18142311)
Supplement: Supplementary file 1 [file cancers-18-02311-s001.zip › Supplementary_Table_S4.pdf]

Supplementary Table S4. Product-stratified 1-year mortality and time-to-next-treatment outcomes after CAR-T cell therapy (ide-cel, cilta-cel) by renal-function stratum.

Outcome	ide-cel	ide-cel	cilta-cel	cilta-cel
	eGFR <30 vs >60	eGFR 30–60 vs >60	eGFR <30 vs >60	eGFR 30–60 vs >60
	(n=76)	(n=260)	(n=157)	(n=402)
**1-Year Mortality HR (95% CI); log-rank p**	0.826 (0.403–1.693); p=0.600	1.081 (0.718–1.627); p=0.710	1.502 (0.887–2.541); p=0.130	1.259 (0.786–2.018); p=0.340
**1-Year Survival probability**	79.8% vs 77.9%	79.9% vs 81.5%	69.9% vs 78.0%	86.9% vs 89.5%
**1-Year TTNT HR (95% CI); log-rank p**	0.778 (0.466–1.298); p=0.500	1.058 (0.797–1.406); p=0.880	1.375 (0.853–2.215); p=0.190	1.057 (0.751–1.487); p=0.750
**1-Year TTNT Event Free probability**	61.4% vs 54.4%	59.1% vs 60.7%	79.0% vs 84.7%	77.1% vs 78.2%

Hazard ratios (95% CI) and log-rank p-values are shown for 1-year mortality and time to next myeloma-directed treatment (TTNT), stratified by individual CAR-T products. Comparisons were performed within each product between patients with eGFR <30 or eGFR 30–60 mL/min/1.73 m² and those with eGFR >60 mL/min/1.73 m². Survival and TTNT event-free probabilities are Kaplan-Meier estimates at 1 year. Bold indicates statistically significant results.
