# Supplementary material for: Efficacy and Safety of CAR-T Cell Therapy and Bispecific Antibodies in Relapsed/Refractory Multiple Myeloma with Renal Impairment: A Propensity Score-Matched Analysis
Source: Cancers (Basel). 2026 Jul 17;18(14):2311. doi: 10.3390/cancers18142311 (PMC13406253; doi:10.3390/cancers18142311)
Supplement: Supplementary file 1 [file cancers-18-02311-s001.zip › Supplementary_Table_S5.pdf]

Supplementary Table S5. Product-stratified 1-year mortality and time-to-next-treatment outcomes after bispecific antibody therapy (teclistamab, talquetamab, elranatamab) by renal-function stratum.

Outcome	Teclistamab	Teclistamab	Talquetamab	Talquetamab	Elranatamab	Elranatamab
	eGFR <30 vs >60	eGFR 30-60 vs >60	eGFR <30 vs >60	eGFR 30-60 vs >60	eGFR <30 vs >60	eGFR 30-60 vs >60
	(n=480)	(n=923)	(n=231)	(n=484)	(n=67)	(n=115)
**1-Year Mortality HR (95% CI); log-rank p**	1.183 (0.921–1.519); p=0.188	1.083 (0.897–1.309); p=0.405	1.280 (0.914–1.792); p=0.149	1.145 (0.890–1.472); p=0.291	1.293 (0.723–2.310); p=0.385	1.134 (0.697–1.844); p=0.612
**1-Year Survival probability**	66.8% vs 71.0%	71.3% vs 72.7%	57.3% vs 64.6%	65.1% vs 68.9%	55.9% vs 60.1%	61.7% vs 64.0%
**1-Year TTNT HR (95% CI); log-rank p**	1.082 (0.885–1.324); p=0.440	0.995 (0.869–1.139); p=0.942	1.223 (0.956–1.565); p=0.108	1.086 (0.918–1.286); p=0.334	1.256 (0.753–2.097); p=0.380	1.054 (0.719–1.546); p=0.787
**1-Year TTNT event free probability**	53.8% vs 55.3%	48.8% vs 47.4%	27.6% vs 37.6%	32.2% vs 34.7%	46.1% vs 54.2%	43.2% vs 42.6%

Hazard ratios (95% CI) and log-rank p-values are shown for 1-year mortality and time to next myeloma-directed treatment (TTNT), stratified by individual bispecific antibody agents. Comparisons were performed within each agent between patients with eGFR <30 or eGFR 30–60 mL/min/1.73 m² and those with eGFR >60 mL/min/1.73 m². Survival and TTNT event-free probabilities are Kaplan-Meier estimates at 1 year (stratum vs. eGFR >60 reference). Bold indicates statistically significant results.
