# Supplementary material for: Efficacy and Safety of CAR-T Cell Therapy and Bispecific Antibodies in Relapsed/Refractory Multiple Myeloma with Renal Impairment: A Propensity Score-Matched Analysis
Source: Cancers (Basel). 2026 Jul 17;18(14):2311. doi: 10.3390/cancers18142311 (PMC13406253; doi:10.3390/cancers18142311)
Supplement: Supplementary file 1 [file cancers-18-02311-s001.zip › Supplementary_Table_S6.pdf]

Supplementary Table S6. Product-stratified safety outcomes after CAR-T cell therapy by renal-function stratum.

Outcome	Time in months	ide-cel	ide-cel	cilta-cel	cilta-cel
		eGFR <30 vs >60	eGFR 30–60 vs >60	eGFR <30 vs >60	eGFR 30–60 vs >60
		(n=76)	(n=260)	(n=157)	(n=402)
**CRS**	1	1.106 (0.876–1.397)	1.060 (0.881–1.275)	0.900 (0.754–1.074)	0.939 (0.829–1.063)
**ICANS**	1	Suppressed	1.481 (0.730–3.007)	1.007 (0.535–1.895)	1.068 (0.734–1.552)
**AKI**	1	0.938 (0.500–1.758)	1.216 (0.816–1.813)	1.571 (0.954–2.589)	1.262 (0.930–1.714)
	3	1.056 (0.602–1.849)	1.262 (0.875–1.820)	1.542 (0.971–2.448)	1.275 (0.961–1.692)
	6	1.056 (0.602–1.849)	1.196 (0.860–1.663)	1.560 (0.995–2.446)	1.272 (0.985–1.642)
**Grade ≥3 Anemia**	1	1.275 (0.978–1.662)	1.076 (0.898–1.289)	**1.477 (1.183–1.844)**	1.126 (0.935–1.356)
	3	1.209 (0.943–1.552)	1.072 (0.902–1.274)	**1.471 (1.189–1.818)**	1.175 (0.987–1.399)

Outcome	Time in months	ide-cel	ide-cel	cilta-cel	cilta-cel
		eGFR <30 vs >60	eGFR 30–60 vs >60	eGFR <30 vs >60	eGFR 30–60 vs >60
		(n=76)	(n=260)	(n=157)	(n=402)
	6	1.182 (0.925–1.510)	1.092 (0.927–1.287)	**1.486 (1.211–1.823)**	1.159 (0.980–1.371)
**Grade ≥3 Thrombocytopenia**	1	1.020 (0.819–1.270)	1.049 (0.902–1.220)	**1.260 (1.021–1.556)**	1.061 (0.889–1.267)
	3	1.038 (0.847–1.271)	1.054 (0.912–1.218)	**1.267 (1.033–1.554)**	1.060 (0.891–1.261)
	6	1.057 (0.865–1.291)	1.053 (0.914–1.213)	**1.234 (1.009–1.509)**	1.070 (0.905–1.265)
**Grade ≥3 Neutropenia**	1	0.985 (0.867–1.119)	1.018 (0.955–1.084)	1.047 (0.953–1.150)	1.026 (0.976–1.078)
	3	0.970 (0.857–1.098)	1.017 (0.958–1.080)	1.038 (0.949–1.136)	1.028 (0.981–1.078)
	6	0.956 (0.847–1.078)	1.017 (0.960–1.078)	1.038 (0.951–1.133)	1.028 (0.982–1.076)
**Infections (all-grade)**	1	1.091 (0.673–1.769)	1.121 (0.823–1.527)	1.294 (0.878–1.907)	1.088 (0.849–1.395)

Outcome	Time in months	ide-cel	ide-cel	cilta-cel	cilta-cel
		eGFR <30 vs >60	eGFR 30–60 vs >60	eGFR <30 vs >60	eGFR 30–60 vs >60
		(n=76)	(n=260)	(n=157)	(n=402)
	3	1.000 (0.682–1.467)	1.114 (0.867–1.431)	1.349 (0.974–1.867)	1.065 (0.869–1.305)
	6	1.059 (0.750–1.494)	1.097 (0.878–1.369)	1.268 (0.900–1.787)	1.048 (0.876–1.254)
**Hypogammaglobulinemia**	1	0.828 (0.534–1.282)	0.947 (0.719–1.246)	0.889 (0.647–1.222)	0.957 (0.791–1.159)
	3	0.944 (0.669–1.333)	0.933 (0.751–1.158)	1.043 (0.820–1.326)	1.019 (0.892–1.164)
	6	1.158 (0.862–1.556)	0.992 (0.823–1.194)	1.065 (0.858–1.322)	0.987 (0.878–1.110)

Risk ratios with 95% confidence intervals are shown for safety outcomes at 1, 3, and 6 months, stratified by individual CAR-T products. Comparisons were performed within each product between patients with eGFR <30 or eGFR 30–60 mL/min/1.73 m² and those with eGFR >60 mL/min/1.73 m². CRS, ICANS and AKI were assessed at 1 month only. Suppressed indicates small cell counts withheld by the TriNetX platform. Bold indicates statistically significant results.
