# Supplementary material for: Efficacy and Safety of CAR-T Cell Therapy and Bispecific Antibodies in Relapsed/Refractory Multiple Myeloma with Renal Impairment: A Propensity Score-Matched Analysis
Source: Cancers (Basel). 2026 Jul 17;18(14):2311. doi: 10.3390/cancers18142311 (PMC13406253; doi:10.3390/cancers18142311)
Supplement: Supplementary file 1 [file cancers-18-02311-s001.zip › Supplementary_Table_S7.pdf]

Supplementary Table S7. Product-stratified safety outcomes after bispecific antibody therapy by renal-function stratum.

Outcome	Time in months	Teclistamab	Teclistamab	Talquetamab	Talquetamab	Elranatamab	Elranatamab
		**eGFR <30 vs >60 (n=480)**	**eGFR 30-60 vs >60 (n=923)**	**eGFR <30 vs >60 (n=231)**	**eGFR 30-60 vs >60 (n=484)**	**eGFR <30 vs >60 (n=67)**	**eGFR 30-60 vs >60 (n=115)**
**CRS**	1	0.70 (0.52–0.93)	0.93 (0.76–1.13)	0.87 (0.62–1.23)	0.96 (0.77–1.20)	Suppressed	0.84 (0.50–1.41)
**ICANS**	1	0.93 (0.44–1.96)	0.87 (0.52–1.45)	Suppressed	1.11 (0.60–2.03)	Suppressed	Suppressed
**AKI**	1	**2.36 (1.83–3.06)**	**1.81 (1.45–2.24)**	**2.08 (1.48–2.93)**	**1.49 (1.14–1.95)**	1.33 (0.68–2.60)	1.64 (0.89–3.03)
**Grade ≥3 Anemia**	1	**1.42 (1.22–1.66)**	1.12 (0.99–1.27)	**1.33 (1.09–1.61)**	**1.21 (1.03–1.42)**	1.23 (0.88–1.74)	1.29 (0.90–1.84)
	3	**1.39 (1.20–1.60)**	1.11 (0.99–1.25)	**1.31 (1.10–1.55)**	**1.15 (1.01–1.32)**	1.28 (0.94–1.76)	1.30 (0.94–1.79)
	6	**1.38 (1.21–1.58)**	1.11 (1.00–1.24)	**1.20 (1.03–1.40)**	1.14 (1.00–1.29)	1.24 (0.92–1.67)	1.20 (0.89–1.62)
**Grade ≥3 Thrombocytopenia**	1	**1.28 (1.01–1.63)**	1.15 (0.96–1.37)	**1.40 (1.07–1.83)**	1.16 (0.95–1.43)	1.18 (0.75–1.86)	1.17 (0.77–1.79)
	3	**1.28 (1.03–1.58)**	1.13 (0.97–1.32)	**1.34 (1.05–1.70)**	1.14 (0.95–1.36)	1.20 (0.80–1.81)	0.93 (0.66–1.31)
	6	**1.29 (1.05–1.59)**	1.13 (0.97–1.31)	1.08 (0.90–1.31)	1.11 (0.94–1.31)	1.28 (0.86–1.91)	1.14 (0.79–1.66)

Outcome	Time in months	Teclistamab	Teclistamab	Talquetamab	Talquetamab	Elranatamab	Elranatamab
		eGFR <30 vs >60 (n=480)	eGFR 30-60 vs >60 (n=923)	eGFR <30 vs >60 (n=231)	eGFR 30-60 vs >60 (n=484)	eGFR <30 vs >60 (n=67)	eGFR 30-60 vs >60 (n=115)
**Grade ≥3 Neutropenia**	1	1.00 (0.78–1.28)	1.01 (0.85–1.20)	1.05 (0.77–1.43)	1.16 (0.93–1.44)	0.77 (0.40–1.45)	1.00 (0.62–1.61)
	3	0.99 (0.82–1.19)	1.00 (0.88–1.14)	0.99 (0.79–1.24)	1.11 (0.95–1.29)	0.84 (0.52–1.35)	0.95 (0.66–1.37)
	6	0.97 (0.83–1.13)	0.98 (0.87–1.10)	1.25 (1.01–1.55)	1.09 (0.95–1.24)	0.96 (0.63–1.46)	1.00 (0.74–1.35)
**Infections (all-grade)**	1	**1.30 (1.01–1.67)**	1.01 (0.83–1.23)	1.15 (0.86–1.55)	1.19 (0.96–1.48)	0.87 (0.45–1.68)	1.00 (0.61–1.63)
	3	1.05 (0.87–1.25)	0.97 (0.84–1.11)	1.10 (0.86–1.42)	1.10 (0.92–1.32)	1.04 (0.68–1.60)	1.16 (0.78–1.72)
	6	1.09 (0.93–1.27)	0.99 (0.88–1.11)	1.04 (0.84–1.30)	1.07 (0.91–1.26)	0.94 (0.64–1.36)	0.96 (0.72–1.29)
**Hypogammaglobulinemia**	1	1.19 (0.96–1.46)	1.09 (0.94–1.27)	0.93 (0.69–1.24)	0.97 (0.80–1.17)	1.00 (0.50–1.99)	0.97 (0.63–1.49)
	3	1.10 (0.94–1.29)	1.04 (0.92–1.17)	0.82 (0.64–1.04)	0.93 (0.80–1.08)	0.95 (0.55–1.64)	0.98 (0.72–1.34)
	6	1.08 (0.93–1.25)	1.02 (0.91–1.13)	0.91 (0.73–1.12)	0.97 (0.85–1.10)	0.92 (0.57–1.47)	1.02 (0.77–1.36)

Risk ratios with 95% confidence intervals are shown for safety outcomes at 1, 3, and 6 months, stratified by individual bispecific antibody agents. Comparisons were performed within each agent between patients with eGFR <30 or eGFR 30–60 mL/min/1.73 m² and those with eGFR >60 mL/min/1.73 m². CRS, ICANS and AKI were assessed at 1 month only. Suppressed indicates small cell counts withheld by the TriNetX platform. Bold indicates statistically significant results.
