# Supplementary material for: Efficacy and Safety of CAR-T Cell Therapy and Bispecific Antibodies in Relapsed/Refractory Multiple Myeloma with Renal Impairment: A Propensity Score-Matched Analysis
Source: Cancers (Basel). 2026 Jul 17;18(14):2311. doi: 10.3390/cancers18142311 (PMC13406253; doi:10.3390/cancers18142311)
Supplement: Supplementary file 1 [file cancers-18-02311-s001.zip › Supplementary_Table_S8.pdf]

Supplementary Table S8. Benjamini–Hochberg False Discovery Rate (FDR)–Adjusted Safety Outcome p-Values.

All 72 statistical comparisons reported in Table 4 (safety outcomes across CRS, ICANS, AKI, grade ≥ 3 anemia, grade ≥ 3 thrombocytopenia, grade ≥ 3 neutropenia, infections, and hypogammaglobulinemia, at 1, 3, and 6 months, across four renal-function comparisons) were adjusted for multiple testing using the Benjamini–Hochberg procedure to control the false discovery rate at 5%. Raw p-values were ranked from smallest to largest and each was compared against (rank/72) × 0.05; adjusted q-values are reported. Comparisons with q < 0.05 are considered to survive correction for multiple testing and are highlighted.

Outcome	Timepoint	Comparison	Raw p-value	BH-FDR q-value	Survives FDR (q<0.05)
Anemia	1mo	CAR-T Severe RI	<0.001	0.008	Yes
Anemia	1mo	BsAb Severe RI	<0.001	0.008	Yes
AKI	1mo	BsAb Severe RI	<0.001	0.008	Yes
AKI	1mo	BsAb Moderate RI	<0.001	0.008	Yes
Anemia	6mo	CAR-T Severe RI	<0.001	0.008	Yes
Anemia	6mo	BsAb Severe RI	<0.001	0.008	Yes
Anemia	3mo	BsAb Severe RI	<0.001	0.008	Yes
Anemia	3mo	CAR-T Severe RI	<0.001	0.008	Yes
AKI	1mo	CAR-T Moderate RI	0.003	0.024	Yes
Thrombocytopenia	3mo	CAR-T Severe RI	0.004	0.026	Yes
Thrombocytopenia	6mo	CAR-T Severe RI	0.004	0.026	Yes
Anemia	3mo	CAR-T Moderate RI	0.005	0.028	Yes
Anemia	1mo	CAR-T Moderate RI	0.005	0.028	Yes
Thrombocytopenia	6mo	BsAb Severe RI	0.006	0.031	Yes
Anemia	6mo	CAR-T Moderate RI	0.007	0.034	Yes
Thrombocytopenia	1mo	BsAb Severe RI	0.009	0.040	Yes
Infections	1mo	BsAb Severe RI	0.011	0.047	Yes
Thrombocytopenia	3mo	BsAb Severe RI	0.018	0.072	No
Thrombocytopenia	1mo	CAR-T Severe RI	0.020	0.076	No
Anemia	6mo	BsAb Moderate RI	0.026	0.094	No
Anemia	3mo	BsAb Moderate RI	0.031	0.106	No
Hypogammaglobulinemia	6mo	CAR-T Severe RI	0.040	0.131	No
Anemia	1mo	BsAb Moderate RI	0.046	0.144	No
AKI	1mo	CAR-T Severe RI	0.070	0.210	No
Infections	6mo	CAR-T Severe RI	0.080	0.222	No
Infections	3mo	CAR-T Severe RI	0.080	0.222	No
Thrombocytopenia	1mo	BsAb Moderate RI	0.094	0.251	No
Neutropenia	1mo	CAR-T Moderate RI	0.102	0.262	No
Hypogammaglobulinemia	1mo	BsAb Severe RI	0.111	0.276	No
Thrombocytopenia	3mo	BsAb Moderate RI	0.124	0.298	No
Neutropenia	3mo	CAR-T Severe RI	0.140	0.323	No
CRS	1mo	BsAb Moderate RI	0.144	0.323	No
Neutropenia	3mo	CAR-T Moderate RI	0.148	0.323	No
Neutropenia	6mo	CAR-T Moderate RI	0.162	0.333	No
Hypogammaglobulinemia	3mo	CAR-T Severe RI	0.170	0.333	No
Neutropenia	6mo	CAR-T Severe RI	0.170	0.333	No
Thrombocytopenia	6mo	BsAb Moderate RI	0.171	0.333	No
CRS	1mo	BsAb Severe RI	0.222	0.421	No
Infections	6mo	BsAb Severe RI	0.260	0.478	No
Infections	3mo	BsAb Severe RI	0.269	0.478	No
Thrombocytopenia	1mo	CAR-T Moderate RI	0.272	0.478	No
ICANS	1mo	CAR-T Moderate RI	0.309	0.519	No
Neutropenia	1mo	CAR-T Severe RI	0.310	0.519	No
Thrombocytopenia	6mo	CAR-T Moderate RI	0.339	0.532	No
Infections	1mo	CAR-T Severe RI	0.340	0.532	No
Thrombocytopenia	3mo	CAR-T Moderate RI	0.340	0.532	No
Neutropenia	1mo	BsAb Severe RI	0.455	0.682	No
Hypogammaglobulinemia	1mo	CAR-T Moderate RI	0.462	0.682	No
Infections	1mo	BsAb Moderate RI	0.464	0.682	No
ICANS	1mo	CAR-T Severe RI	0.510	0.734	No

Outcome	Timepoint	Comparison	Raw p-value	BH-FDR q-value	Survives FDR (q<0.05)
Infections	6mo	CAR-T Moderate RI	0.551	0.778	No
Neutropenia	6mo	BsAb Severe RI	0.572	0.792	No
Hypogammaglobulinemia	1mo	BsAb Moderate RI	0.595	0.808	No
Infections	3mo	CAR-T Moderate RI	0.678	0.892	No
Hypogammaglobulinemia	3mo	BsAb Moderate RI	0.728	0.892	No
Infections	1mo	CAR-T Moderate RI	0.729	0.892	No
Hypogammaglobulinemia	3mo	BsAb Severe RI	0.729	0.892	No
ICANS	1mo	BsAb Severe RI	0.754	0.892	No
Neutropenia	3mo	BsAb Moderate RI	0.757	0.892	No
Hypogammaglobulinemia	6mo	CAR-T Moderate RI	0.775	0.892	No
Hypogammaglobulinemia	1mo	CAR-T Severe RI	0.780	0.892	No
Infections	3mo	BsAb Moderate RI	0.788	0.892	No
CRS	1mo	CAR-T Severe RI	0.790	0.892	No
Neutropenia	6mo	BsAb Moderate RI	0.799	0.892	No
Neutropenia	3mo	BsAb Severe RI	0.813	0.892	No
Hypogammaglobulinemia	6mo	BsAb Severe RI	0.821	0.892	No
Infections	6mo	BsAb Moderate RI	0.830	0.892	No
Neutropenia	1mo	BsAb Moderate RI	0.879	0.915	No
Hypogammaglobulinemia	3mo	CAR-T Moderate RI	0.885	0.915	No
Hypogammaglobulinemia	6mo	BsAb Moderate RI	0.890	0.915	No
CRS	1mo	CAR-T Moderate RI	0.923	0.936	No
ICANS	1mo	BsAb Moderate RI	1.000	1.000	No

p-values reported in the original Table 4 as "< 0.001" were conservatively treated as 0.0009 for ranking purposes; true q-values for these comparisons may be smaller than shown. AKI, acute kidney injury; BH, Benjamini–Hochberg; CI, confidence interval; CRS, cytokine release syndrome; FDR, false discovery rate; ICANS, immune effector cell–associated neurotoxicity syndrome.
