# Supplementary figures and images for: Efficacy and Safety of CAR-T Cell Therapy and Bispecific Antibodies in Relapsed/Refractory Multiple Myeloma with Renal Impairment: A Propensity Score-Matched Analysis
Source: Cancers (Basel). 2026 Jul 17;18(14):2311. doi: 10.3390/cancers18142311 (PMC13406253; doi:10.3390/cancers18142311)

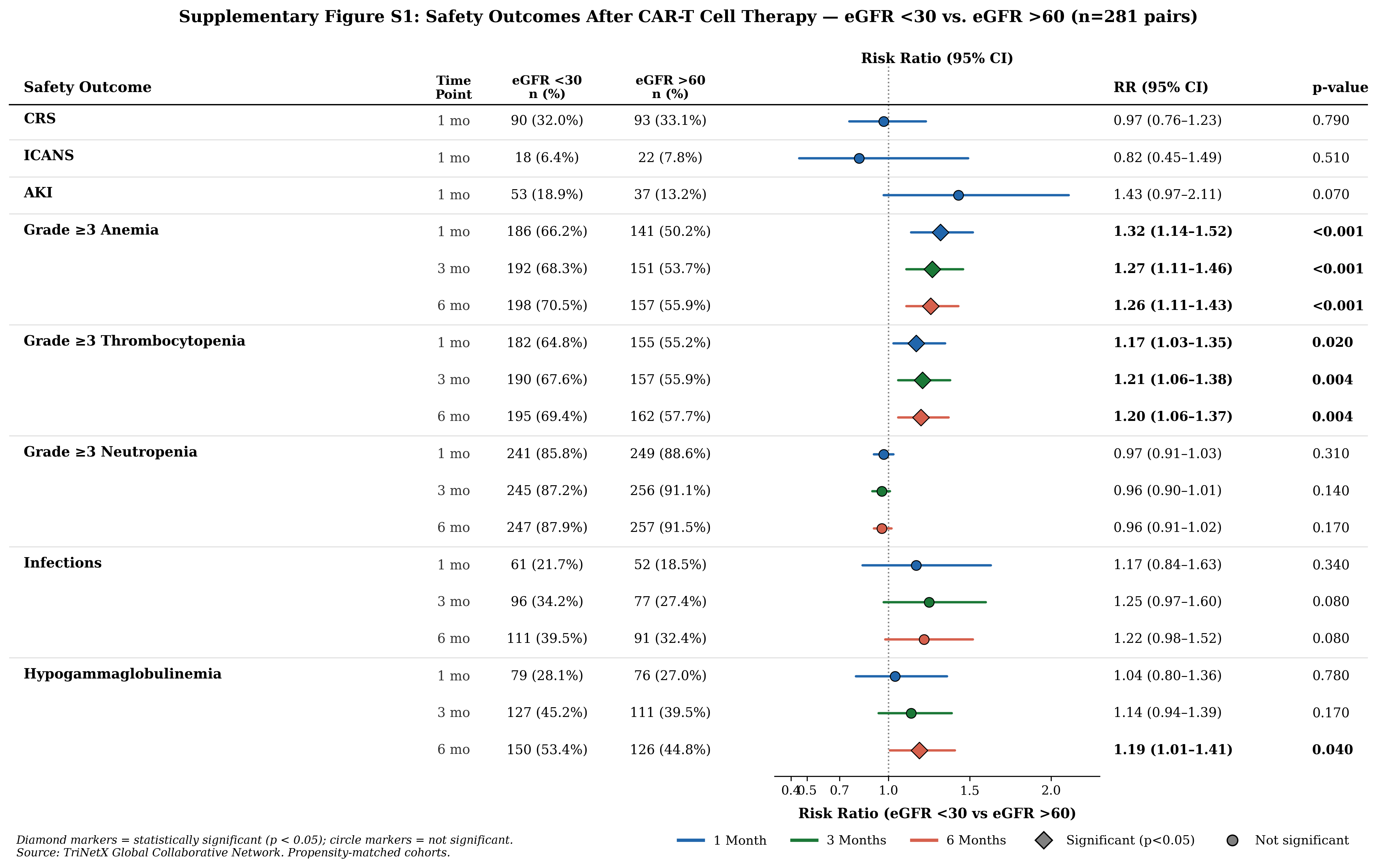

Supplement: Supplementary file 1 [file cancers-18-02311-s001.zip › Supplementary_Figure_S1.png]

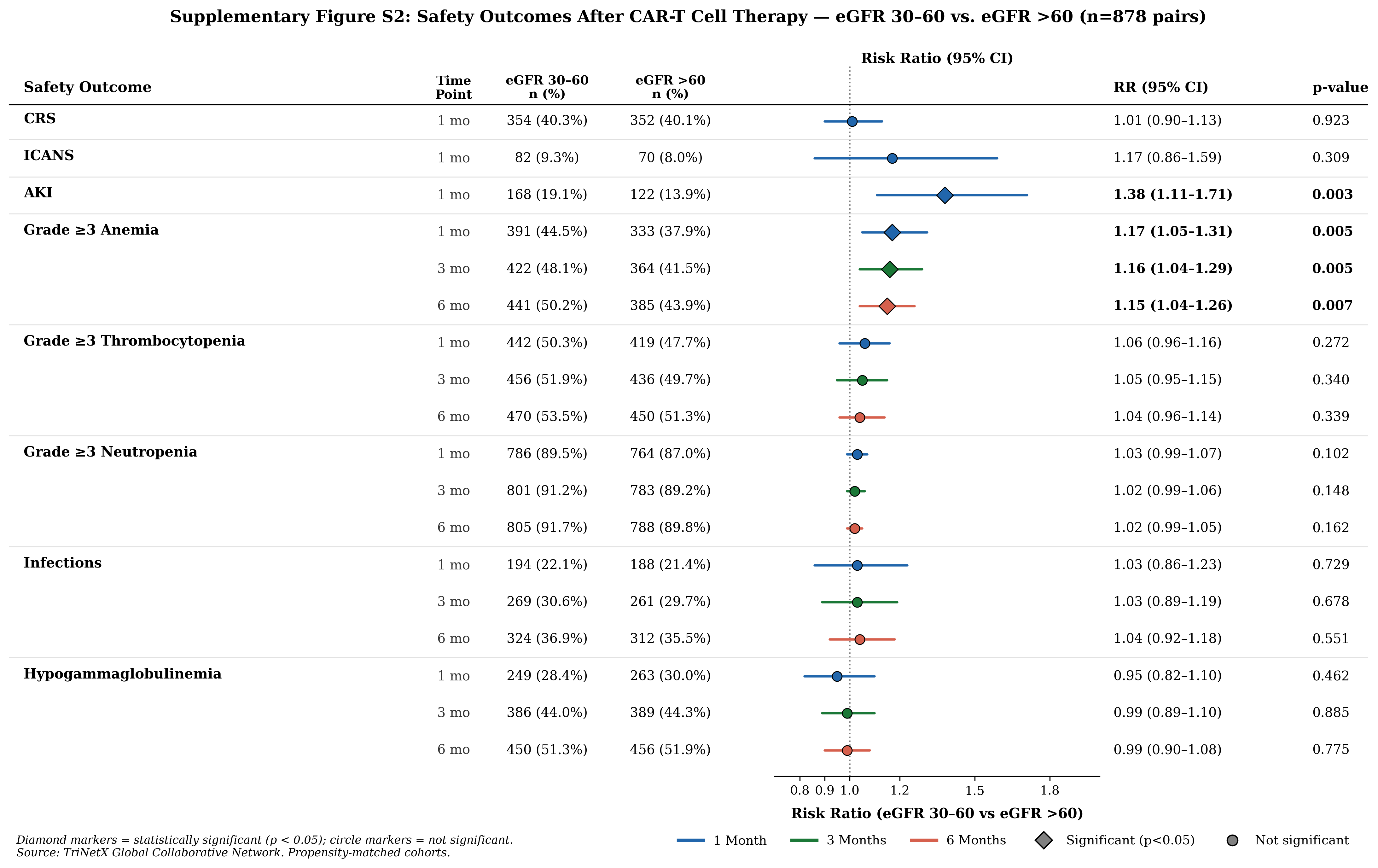

Supplement: Supplementary file 1 [file cancers-18-02311-s001.zip › Supplementary_Figure_S2.png]

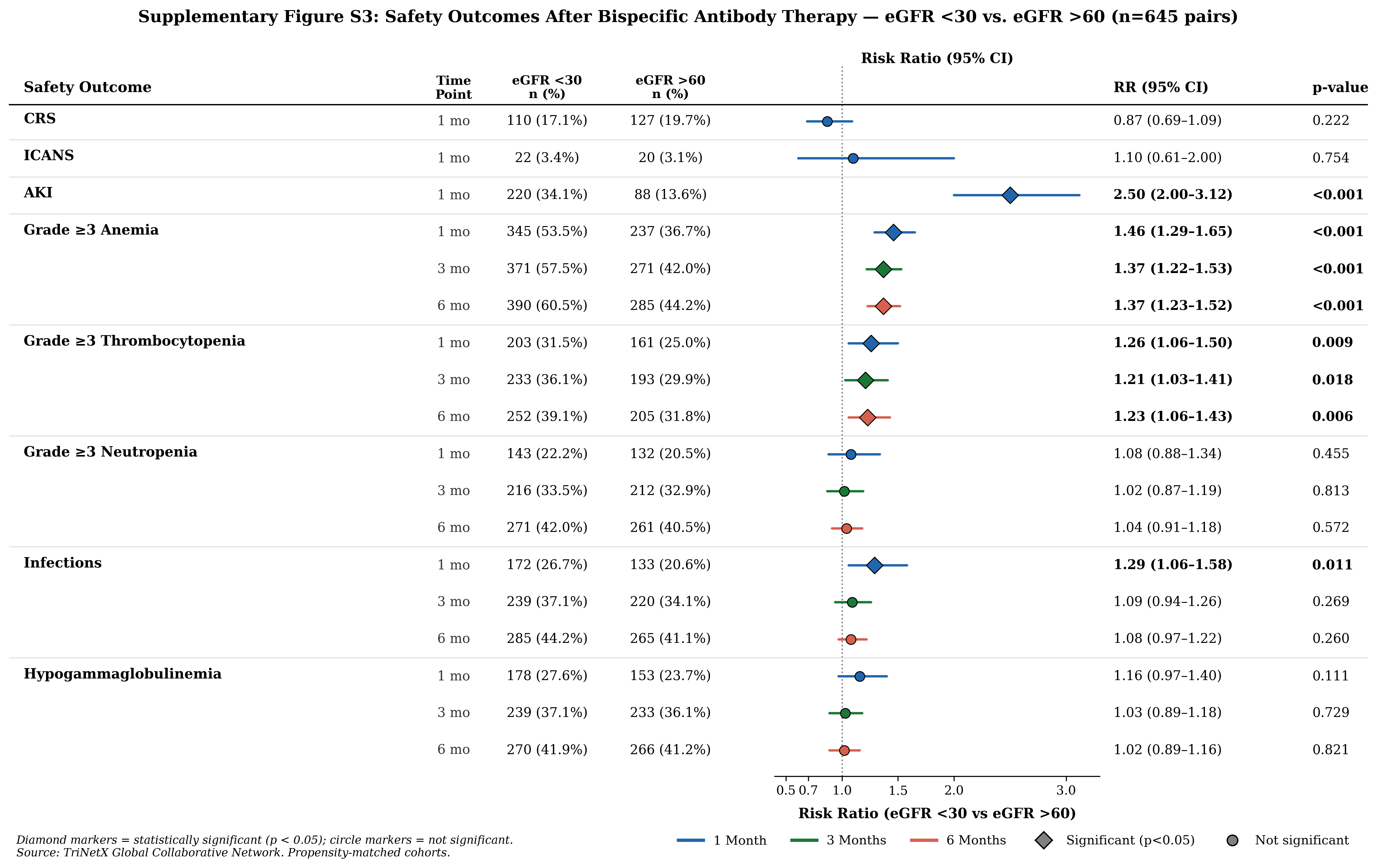

Supplement: Supplementary file 1 [file cancers-18-02311-s001.zip › Supplementary_Figure_S3.png]

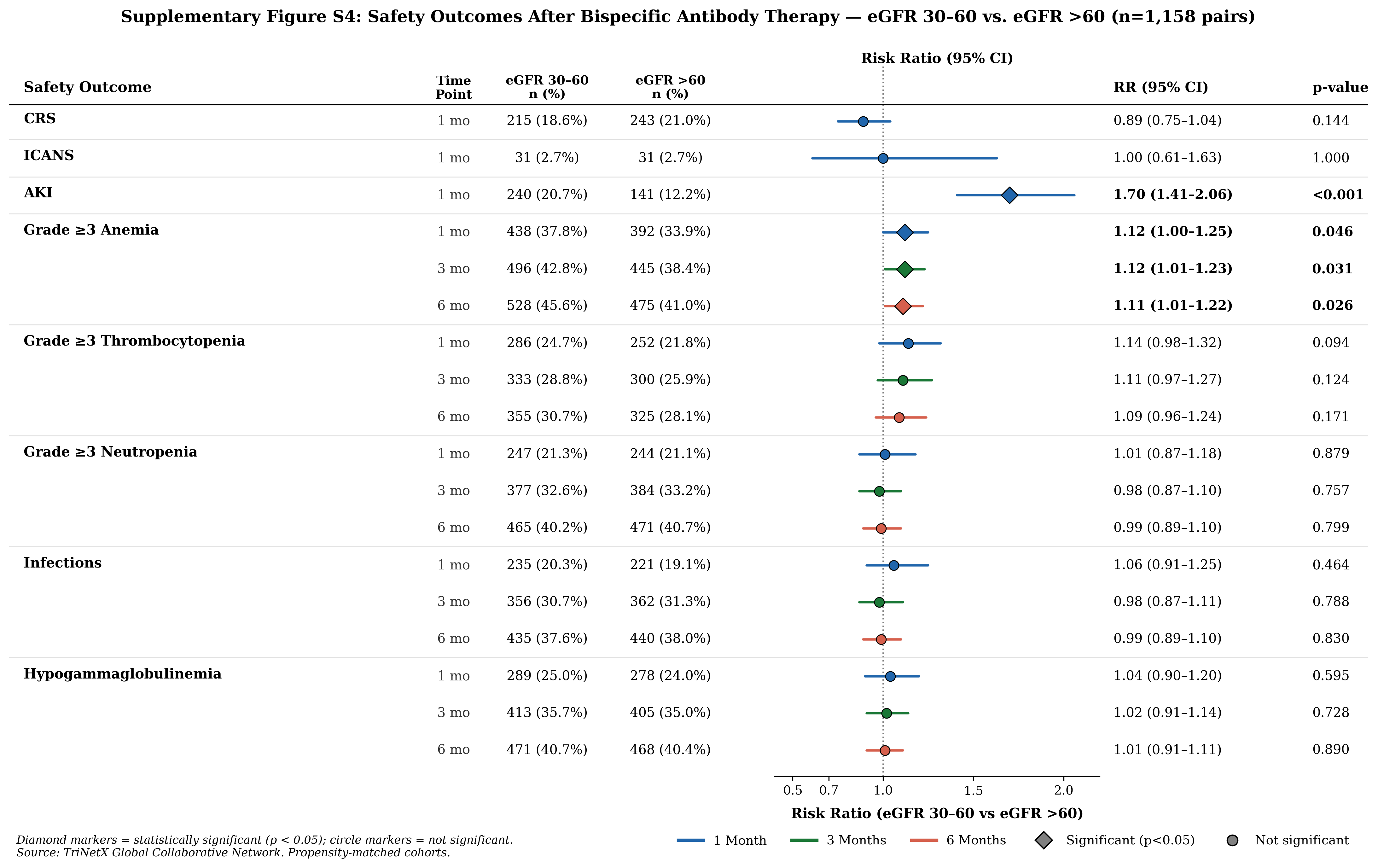

Supplement: Supplementary file 1 [file cancers-18-02311-s001.zip › Supplementary_Figure_S4.png]
